# Conserved Calcineurin A splice variants regulate both constitutive and experience-dependent behaviors through tissue-specific signaling

**DOI:** 10.1371/journal.pgen.1011884

**Published:** 2025-09-26

**Authors:** Martina Rudgalvyte, Dominique A. Glauser

**Affiliations:** Department of Biology, University of Fribourg, Chemin du Musée, Fribourg, Switzerland; Tata Institute of Fundamental Research, INDIA

## Abstract

Calcineurin is a conserved, calcium-regulated phosphatase involved in various functions, including muscle physiology and nervous system activity. The Calcineurin A (CnA) catalytic subunit consists of a phosphatase region and a regulatory domain, which includes a Calcineurin B binding helix, a Calmodulin binding domain, and an auto-inhibitory domain, separated by linker regions (LR1 and LR2). CnA diversity is generated via the existence of paralogous genes and via alternative splicing, producing tissue-specific isoforms, whose functional significance is only partially understood. Our analyses reveal that the LR2 region is an alternative splicing hotspot conserved across paralogous genes and across species, from humans to nematodes. To investigate LR2 variants’ role *in vivo*, we used the *C. elegans* model, where a single gene, *tax-6*, encodes CnA/TAX-6 and produces TAX-6a and TAX-6b/c variants with distinct LR2 regions. TAX-6a is the predominant neuronal isoform, while TAX-6b/c are enriched in muscles. We generated isoform-specific deletions, gain-of-function mutations, and phosphosite mutations using CRISPR/Cas9, and selectively up-regulated Calcineurin signaling in neurons and muscles with transgenes. We quantified behavioral parameters modulated by Calcineurin signaling, such as crawling speed and responses to noxious heat, both as constitutive and experience-dependent traits. Our results show distinct, non-redundant functions for TAX-6a and TAX-6b/c isoforms, synergistic actions across neurons and muscles, and suggest the differential involvement of TAX-6 phosphorylation-based regulation across these tissues in the modulation of phenotypic outcomes. Overall, our study underscores the importance of LR2-affecting splice variants in regulating both constitutive and experience-dependent behaviors and suggests that such tissue-specific regulation might be conserved across species.

## Author summary

Alternative splicing is a widespread mechanism for diversifying isoform sequences encoded by single genes in eukaryotes. Studying the *in vivo* functions of specific isoforms remains challenging due to their partial redundancy and overlapping expression patterns. As a result, gene-level functions are often better understood than those of individual isoforms. Using *C. elegans*, we employed genome editing to create targeted lesions in alternative exons of the Calcineurin A gene *tax-6* and systematically characterized the resulting mutants to uncover distinct roles for specific variants in muscle and the nervous system. Given the conservation of Calcineurin isoforms from worms to humans, our findings offer insights into their functional diversification across species and highlight the power of genome editing for dissecting alternative exon functions in *C. elegans*.

## Introduction

Calcineurin signaling plays a pivotal role in regulating a variety of calcium-dependent processes across eukaryotes, from immune cell activation to muscular tissue homeostasis and neuronal plasticity [1,2]. Calcineurin is a Ca² ⁺ /calmodulin-dependent serine/threonine phosphatase that functions as a heterodimer, consisting of two subunits: the catalytic Calcineurin A (CnA) and the regulatory Calcineurin B (CnB). Upon intracellular calcium concentration elevation, Calcineurin is activated to dephosphorylate specific substrates. CnA consists of a large catalytic domain on the N-terminal side of the protein and a regulatory domain on the C-terminal side. In canonical CnA, the latter domain includes a CnB-binding helix (BBH), a calmodulin-binding domain (CBD), and an autoinhibitory domain (AID). In the absence of Ca² ⁺ /calmodulin, the AID binds to the catalytic domain, inhibiting the enzyme’s activity. Upon intracellular Ca^2+^ concentration elevation, Ca² ⁺ binds to the CnB subunit and Ca² ⁺ /calmodulin binds to CnA via the CBD, causing a conformational change in the complex to release the auto-inhibition and activate the phosphatase [2].

In mammals, CnA isoforms are encoded by three paralogous genes: *PPP3CA*, *PPP3CB*, and *PPP3CC*, coding for CnAα, CnAβ, and CnAγ, respectively. All three genes are expressed at different levels over a broad range of tissues. CnAα is most abundantly expressed in the nervous system. CnAβ is abundantly expressed in the nervous system as well as in skeletal and cardiac muscle. In the brain, neuron-type expression differences were reported for CnAα and CnAβ [3–5]. CnAγ is predominantly expressed in the testes but has also been found in many other tissues, including in muscle. Each CnA paralogous gene encodes multiple variants, resulting from alternative splicing [6–8]. One extreme CnA splice variant, CnAα 16K, lacks both the catalytic domain and the CBD due to the skipping of 9 exons (joining exon 1 with exon 11). This short protein version was found to interact with full-length isoforms and to slightly up-regulate their calcium sensitivity *in vitro* [9]. Beyond this extreme case of massive protein shortening, the other known CnA isoforms retain the core catalytic region and might only subtly differ in their catalytic properties. Isoform-specific sequence variations are mostly seen at their N- and C-terminal extremities, as well as within the regulatory domain. The specific proline-rich (PP) N-terminal region encoded in the *PPP3CB* paralog and shared across most CnAβ splice variants, was recently found to regulate context-dependent subcellular compartmentation of CnAβ isoforms [10]. The alternative C-terminal region of one CnAβ variant, CnAβ1, lacks the canonical AID domain, but alternative 3’ exons in this isoform encode an LAPD domain which achieves auto-inhibition via a different type of interaction with the catalytic domain, resulting in different (in)activation properties [11]. This specific CnAβ1 isoform also present a unique membrane localization sequence, causing the enzyme to attach to internal membranes upon activation [12]. In addition to these relatively large sequence variations in the N- and C-terminal portions of the protein, smaller variations have also been reported to affect the regulatory region. Alternative inclusion of two small exons (human exon 10 and 13, respectively, in the *PPP3CA* gene) affect linker regions (LR) situated up-stream and down-stream of the CBD, respectively, which we will refer to as LR1 and LR2. Alternative exon 10 inclusion lengthen the LR1 linker between the BBH and the CBD by 9 amino acids. Alternative exon 13 inclusion lengthen the LR2 linker between the CBD and the AID by 19 amino acids. Remarkably, alternative splicing at these locations are conserved across the three paralogs with LR1 and LR2 extending sequences being nearly identical (S1 Fig.). Coordinated shifts in splicing patterns in these regions were reported for all three paralogs during postnatal skeletal muscle development, and this shift was essential for the proper maturation of the muscle fiber function [13]. Whether LR1 and LR2-affecting alternative splicing is conserved across species and whether it affects additional physiological processes in other tissues was largely unknown.

**Fig 1:**
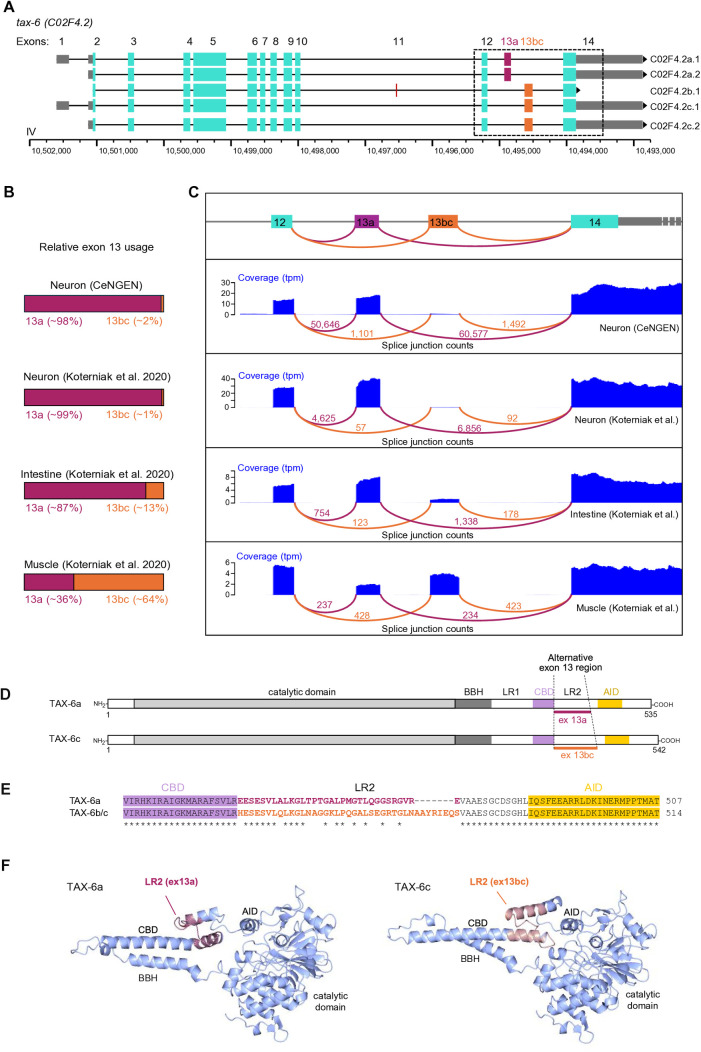
Tissue-specific expression of splice variants diversifying the LR2 region of Calcineurin A/TAX-6 in *C. elegans.* **(A)** *tax-6* transcript variants; graphical presentation showing exon count definition, exon topology, genomic coordinates, and wormbase (WS292) transcript nomenclature (right labels). Constitutive exons in cyan, with untranslated regions in grey; alternative exons in warm colors. Note the decreasing genomic coordinates. Exons 13a and 13bc are mutually exclusive exons. **(B)** Fraction of transcripts using exon 13a or exon 13bc in different tissues based on the mentioned datasets and showing distinct patterns of exon 13 selection in neurons, muscle, and intestine. **(C)** Graphical representation of raw coverage data and splice junction counts from the same datasets as in panel **B. (D)** Protein topology comparison between TAX-6a and TAX-6c proteins highlighting the variable region encoded by alternative exons 13a and 13bc. BBH: Calcineurin B binding helix; LR1: Linker region 1; CBD: Calmodulin binding domain; LR2: Linker Region 2; AID: Auto-inhibitory domain. **(E)** Protein sequence alignment comparing the region affected by alternative splicing in TAX-6a and TAX-6b/c. The colored amino acid font in the LR2 highlights the regions encoded by exons 13a and 13bc respectively. **(F)** AlphaFold structure prediction for TAX-6a and TAX-6c (residues from 43 to 500) highlighting the variable region in the LR2 with warm colors. The N-term and C-term regions with very low prediction scores, as well as the regulatory subunit Calcineurin B, are not depicted. Accessions: TAX-6a: AF-A8X6D8-F1-v4; TAX-6c: AF-Q0G819-F1-v4.

With increasing knowledge about the tissue-specific expression patterns of transcript isoforms and powerful genetic tools to manipulate their expression, *C. elegans* represents an attractive model for studying the relationship between expression and function across alternatively spliced gene isoforms [14–22]. The *C. elegans* genome contains single homologs for CnA and CnB genes, named *tax-6* and *cnb-1,* respectively [23,24]. The TAX-6 protein displays significant sequence homology and shares its general topology with canonical mammalian CnA, with a N-terminal catalytic region and a C-terminal regulatory region comprising BBH, CBD and AID regions as well as linkers between them (Fig 1D). Several studies have examined the role of Calcineurin signaling in *C. elegans*, notably using *tax-6(p675)* loss-of-function mutants or *tax-6(jh107)* gain-of-function mutants, which encode an overactive truncated protein lacking the AID. Loss of *tax-6* leads to thermotaxis abnormalities, increased osmosensation, as well as up-regulated adaptation in the olfactory system [25]. *tax-6* gain-of-function mutants display abnormal enteric muscle contraction [26], larger brood size and increased sensitivity to serotonin [27]. A recent study has also demonstrated that Calcineurin signaling plays a role in modulating reversal responses triggered by noxious heat stimuli. Specifically, when Calcineurin is up-regulated, it increases the responsiveness of naïve animals to noxious heat stimuli [28]. Additionally, experimental manipulations that either enhance or reduce Calcineurin signaling were shown to block the normal adaptation to thermo-nociceptive stimuli. In wild-type animals, this adaptation manifests as a progressive decrease in response after repeated heat stimulations over the course of an hour, but such adaptations are impaired when Calcineurin signaling is altered [28].

Five splice variants have been reported so far for *tax-6*, resulting from (i) alternative transcriptional start site, causing a long or a short version of the 5’UTR with alternative exon 1, (ii) alternative inclusion of the 9-nucleotide-long exon 11, and (iii) the mutually exclusive inclusion of two forms of exon 13 (Fig 1A). Transcript *b* is the only one incorporating the small exon 11. *a1* and *a2* transcripts include the most 5’ of the two alternate exon 13, which we will name exon 13a. *b, c1*and *c2* transcripts include the most 3’ of the two alternate exon 13, which we will name exon 13bc. Interestingly, alternative exon 13 of the *C. elegans tax-6* gene codes for most of the LR2 of TAX-6 protein (Fig 1D), which is reminiscent of the situation in human CnA paralogs. While isoform-specific functions for *C. elegans tax-6* are not known, it represents an attractive model in which the function of Calcineurin isoforms can be studied *in vivo* with genetic manipulations.

The goal of the present study was to analyze the expression and function of *tax-6* splice variants affecting the LR2 region of the TAX-6 protein. Detailed analysis of available alternative exon expression datasets in various species indicate a strong conservation of alternative splicing in the LR2 region of CnA in human, mice, zebrafish and all the nematode species examined. We found that, in *C. elegans*, the short LR2 version of TAX-6 (TAX-6a) is the most predominant isoform expressed in neurons, while the long LR2 versions (TAX-6b/c) are enriched in muscle. By combining a series of CRISPR/Cas9-mediated precise deletions/mutations at the endogenous *C. elegans tax-6* locus with behavioral quantification of thermo-nociceptive response and animal locomotion, we could further dissect the functional contribution of TAX-6 isoforms, of phosphosites present in either variant, and of specific tissues via tissue-specific expression of truncated (gain-of-function) mutant proteins. Overall, our data reveal that TAX-6 isoforms are not strictly interchangeable and suggest they undergo differential phosphorylation-dependent regulation in the nervous system and in muscles, from where they regulate various behaviors. Given the extremely high conservation of this pathway and alternative splicing in the LR2 region, we speculate that similar regulation is relevant in other species.

## Results and discussion

### Alternative splicing affecting the LR2 region of CnA is a widespread process found in vertebrates and invertebrates

As described above, the LR2 sequence of CnA is diversified by alternative exon 13 inclusion in mammals and by mutually exclusive alternative exons 13a and 13bc in *C. elegans* (Fig 1A). In each case a long and a short LR2 region is produced in different variants (Fig 1D, E and F). In mammals, exon 13 encodes 10 residues, with a perfectly conserved ATVEAIEADE sequence across paralogous human genes as well as in mice (S1 Fig.). In *C. elegans*, exon 13bc is 21 nucleotides longer than exon 13a, and the LR2 regions in TAX-6b/c is 7 residues longer than that in TAX-6a (S2 Fig). Of note, the homology between exon 13a and 13bc-encoded sequence is very high, such that the most salient difference is the shortening of the LR2 on the C-terminal side in TAX-6a as compared to TAX-6b/c. Remarkably, protein sequence comparison across mammals and worms shows that the variable regions align (S1A Fig.). Therefore, despite a different alternative splicing event type is engaged (exon inclusion/skipping versus mutually exclusive exons) the resulting variations in both worm and mammalian CnA proteins affect a very specific location in the LR2 region. To assess how widespread this process is, we expanded our sequence analysis to include other species. First, we examined the CnA orthologous genes in four other nematode species. Despite up to several hundred million years of separate evolution [29] and variable gene structures, splice variants were identified affecting the exact same protein region (S2 Fig). Likewise, we found isoforms diverging at the same region in zebrafish (S1B Fig). Overall, we didn’t detect obvious sequence motifs in the variable regions that would be shared across mammals, fish and nematodes. Therefore, we conclude that, whereas the sequences of LR2 linker variable regions vary quite broadly, the existence of a long and a short LR2 version is a widespread phenomenon. This observation leads us to speculate that there is a fundamental benefit brought by the existence of CnA variants with variable LR2 regions, and that alternative splicing has either been conserved or convergently evolved to create such variants.

### Tissue-specific TAX-6/CnA isoform expression pattern in *C. elegans*

For the rest of the study, we focused on *C. elegans* TAX-6/CnA variants in the aim of characterizing LR2 variant-specific expression, function and regulation. We started by examining the tissular expression pattern of *tax-*6 alternate exon 13a and 13bc. We considered two previously acquired datasets where alternative exons had been broadly quantified via RNA-seq based approaches: one from the CeNGEN consortium [22,30] and one published by Koterniak and collaborators [20]. Both datasets converged to show that the nervous system almost exclusively expresses exon 13a (representing at least 98% of detected transcripts) (Fig 1B and C). While exon 13a was also predominant in the intestine (~87%), it contributed only 36% of muscular transcripts. The majority (~65%) of the muscular transcripts contained exon 13bc. When examining individual neuronal cell types in the CeNGEN dataset, exon 13a-containing transcripts were systematically expressed at higher levels than exon 13bc-containing transcripts (S3 Fig.). We conclude that neurons and the intestine almost exclusively produce exon 13a-containing isoforms, whereas muscles display a marked enrichment for exon 13bc-containing isoforms.

### Selective loss of *tax-6* Ex13a or Ex13bc reveals partly non-redundant isoform functions

**Fig 2:**
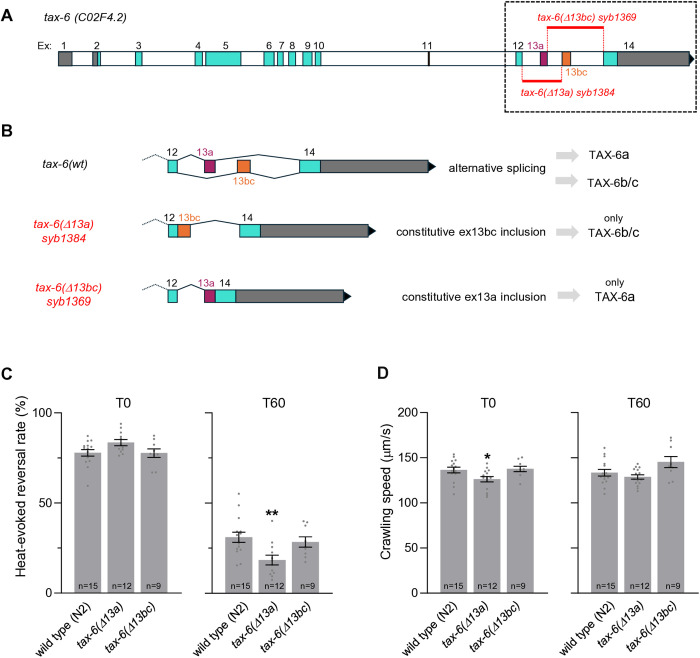
Deletion of *tax-6* alternative exon 13a enhances thermo-nociceptive adaptation and impairs locomotion. **(A)** Schematic representation of the *tax-6* genomic locus with description of the **tax-6(*Δ*13a) syb1369** and **tax-6(*Δ*13bc) syb1384** alleles. **(B)** Predicted impact of mutation on spliced transcripts and TAX-6 variant expression in these mutants. **(C)** Heat-evoked reversal scored in the indicated genotypes. Results as fraction of reversing animals. Each point corresponds to one assay scoring at least 50 animals. Average (grey bars) and s.e.m. (error bars) with indicated n representing the number of independent assays. T0: scoring of naïve animals; T60: scoring of animals exposed to repeated heat stimulation for 60 min. **(D)** Crawling speed measured in the absence of any heat stimulation and reported as under **C.** *, *p* < .05 versus N2(WT) control by Bonferroni-Holm post-hoc tests.

In order to address the biological role of exon 13a and exon 13bc-containing *tax-6* transcripts, we next engineered the *tax-6* endogenous locus using CRISPR/Cas9 to create lines lacking either exon 13a or 13bc (Fig 2A). In the **tax-6(*Δ*13a)** line (allele *syb1384*), we deleted exon 13a as well as upstream and downstream intronic sequences, such that exon 12 and exon 13bc are merged to form a single exon (Fig 2A). This line can only express exon 13bc-containing isoforms (TAX-6b/c), which will therefore replace exon 13a-containing isoforms in tissues where they are normally expressed (such as neurons; see Fig 2B). In the **tax-6(*Δ*13bc)** line (allele *syb1369*), we deleted exon 13bc as well as up-stream and downstream intronic sequences, such that exon 13a and exon 14 are merged (Fig 2A). This line is predicted to express only 13a-containing isoforms (TAX-6a), including in tissues where exon 13bc-containing isoforms are normally expressed (such as muscle. Fig 2B). Although the engineered mutations were designed to minimally affect the endogenous locus, we cannot rule out the possibility that they may cause further unexpected alterations in *tax-6* transcription across tissues. This should be kept in mind when interpreting the results of phenotypic analyses.

To assess the phenotypic impact of these genetic manipulations, we made two types of measures. First, we quantified heat-evoked reversal response in naïve animals (T0) and animals adapted after 60 min of repeated noxious heat stimulation (T60) [31,32]. We previously reported that a *tax-6(j107)* gain-of-function mutation (referred to *tax-6(gf)*) could slightly enhance the response of naïve animals and strongly inhibit adaptation to repeated noxious heat stimuli (see [28] and replication data in S4 Fig.). Very elevated spontaneous reversal rate in the *tax-6(p675)* loss-of-function mutants (as reported in [28]) prevented the assessment of heat-evoked reversal. Therefore, we considered another phenotype and measured crawling speed (baseline locomotion speed in the absence of heat stimuli). Crawling speed of wild type animals was unaffected by the repeated heat-stimulation treatment in wild type (compare crawling speed in T0 and T60 conditions in S4 Fig.). Crawling speed was constitutively reduced in *tax-6(gf)* mutants both at T0 and T60. A similar speed reduction was observed in *tax-6(p675)* loss-of-function mutants at T0*,* but the speed was further reduced at T60 (S4 Fig.). For the rest of study, we therefore quantified both heat-evoked reversal and crawling speed at T0 and T60, as both parameters can reflect perturbations in Calcineurin signaling.

We found that heat-evoked reversal and crawling speed in **tax-6(*Δ*13bc)** mutants were similar to those of wild type, but these parameters were significantly different in **tax-6(*Δ*13a)** mutants (Fig 2C and D). Indeed, the loss of exon 13a in **tax-6(*Δ*13a)** slightly reduced noxious heat-evoked reversal response at T60, but not in naïve animals at T0, which we interpret as an enhancement of thermonociceptive adaptation. Furthermore, the **tax-6(*Δ*13a)** mutants crawled significantly slower than wild-type at T0, but at the same speed at T60 (Fig 2D). Collectively, these results show that animals, which can only express the exon 13bc-containing muscular version of TAX-6, TAX-6b/c, display an enhanced thermo-nociceptive adaptation and dysregulated locomotion. Thus, even if the phenotypic impact of alternative exon deletion is very mild in comparison to that of strong loss- and gain-of-function *tax-6* alleles (Fig 2C and D, S4 Fig.), our observations indicate that exon 13a and exon 13bc of *tax-6* are not entirely interchangeable. In particular, our results imply that TAX-6b/c isoforms cannot fully compensate for the lack of TAX-6a isoform. Of note, an enhanced adaptation of heat-evoked reversal response in **tax-6(*Δ*13a)** mutants is a quite peculiar phenotype. Indeed, the opposite effect (inhibited adaptation) was observed both following TAX-6 overactivation in *tax-6(gf)* mutant (S4 Fig.) and following TAX-6 inhibition with Cyclosporin A treatment [28], although off-target drug effects could not be ruled out in the latter case. Therefore, we propose that the specific features of the TAX-6a isoforms are needed in the nervous system for a proper fine-regulation of Calcineurin signaling activity.

### Isoform-specific truncation mutations produce different phenotypic outcomes

**Fig 3:**
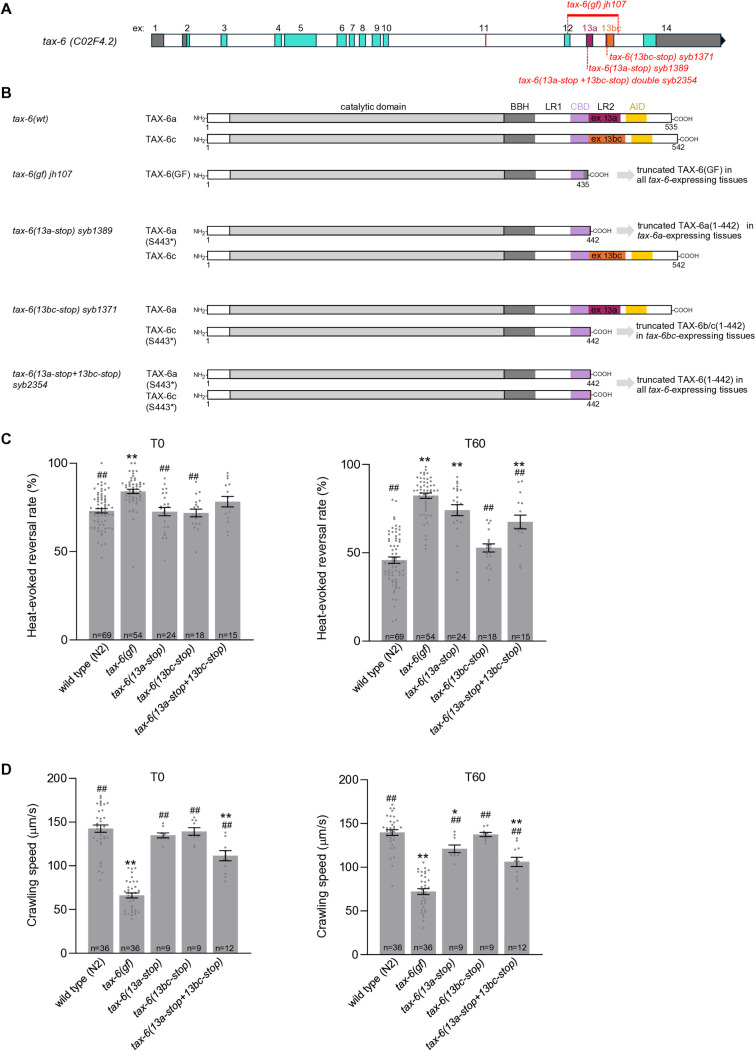
Isoform-specific effect of TAX-6 truncation mutations on thermo-nociceptive responses and locomotion. **(A)** Schematic representation of the *tax-6* genomic locus with description of the *tax-6(13a-stop) syb1389, tax-6(13bc-stop) syb1371* and combined *tax-6(13a-stop+13bc-stop) syb2354* alleles. **(B)** Predicted impact of mutations and truncated TAX-6 variant expression in these mutants. **(C)** Heat-evoked reversal scored in the indicated genotypes. Results as fraction of reversing animals. Each point corresponds to one assay scoring at least 50 animals. Average (grey bars) and s.e.m. (error bars) with indicated n representing the number of independent assays. T0: scoring of naïve animals; T60: scoring of animals exposed to repeated heat stimulation for 60 min. **(D)** Crawling speed measured in the absence of any heat stimulation and reported as under **C.** **, *p* < .01 versus N2(WT); ##, *p* < .01 and #, *p* < .05 versus *tax-6(gf)* by Bonferroni-Holm post-hoc tests.

The *tax-6(gf)* mutation is a deletion covering the end of exon 12, exon 13a and 13bc, and causing a frame shift. The predicted mutant protein only includes the first 435 amino acids of TAX-6 thus lacking the entire LR2 and AID regions (Fig 3A and 3B). Such truncated proteins are expected to be constitutively expressed in every *tax-6*-expressing tissue (Fig 3B). The marked behavioral phenotype observed in this mutant could in principle be caused by a broad overaction of TAX-6 in all tissues where *tax-6* is transcribed, or by its overactivation in more restrained set of tissues, like, e.g., in the nervous system. We reasoned that, by introducing a nonsense S443-to-Stop mutation at the beginning of either exon 13a or exon 13bc, we could create lines where a similar protein truncation is produced in an isoform-specific manner. TAX-6 signaling would therefore be selectively overactivated in either exon 13a-expressing tissues or in exon 13bc-expressing tissues. We thus created a *tax-6(13a-stop)* and a *tax-6(13bc-stop)* line, as well as a *tax-6(13a-stop 13bc-stop)* line affecting both isoforms, and therefore expected to resemble the *tax-6(gf)* mutant (Fig 3A and B).

**Fig 4:**
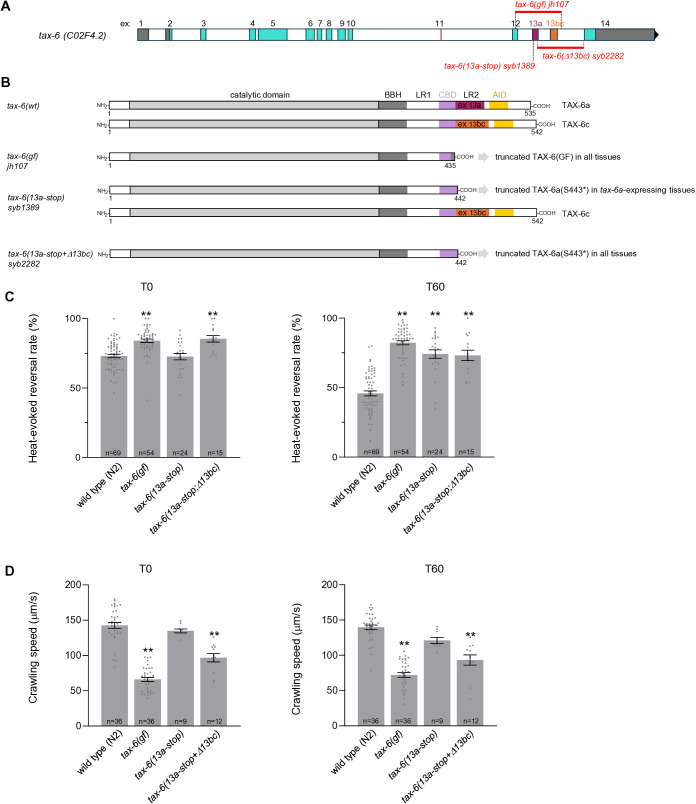
Gain-of-function phenotypes in mutants expressing only truncated TAX-6a. **(A)** Schematic representation of the *tax-6* genomic locus with description of the *tax-6(13a-stop) syb1389, tax-6(13bc-stop) syb1371* and combined *tax-6(13a-stop; 13bc-stop) syb2354* alleles. **(B)** Predicted impact of mutations and truncated TAX-6 variant expression in these mutants. **(C)** Heat-evoked reversal scored in the indicated genotypes. Results as fraction of reversing animals. Each point corresponds to one assay scoring at least 50 animals. Average (grey bars) and s.e.m. (error bars) with indicated n representing the number of independent assays. T0: scoring of naïve animals; T60: scoring of animals exposed to repeated heat stimulation for 60 min. **(D)** Crawling speed measured in the absence of any heat stimulation and reported as under **C.** **, *p* < .01 versus N2(WT); ##, *p* < .01 #, *p* < .05 *tax-6(gf)* by Bonferroni-Holm post-hoc tests. Wild type and *tax-6(gf)* data are replicated from Fig 3.

At T0, none of the single S443* mutations affected heat-evoked reversal, and the slight reversal rate elevation seen in the double mutant as compared to wild type did not reach statistical significance (Fig 3C, left). At T60, the *13bc-stop* mutation had no effect, but the *13a-stop* mutation, alone or in combination with the *13bc-stop* mutation significantly inhibited thermo-nociceptive adaptation (increased reversal rate compared to wild type, Fig 3C, right). This latter phenotype was qualitatively similar to that in the *tax-6(gf)* mutant, but the magnitude of the effect was less pronounced. Regarding speed, we found that the *13bc-stop* mutation had no effect on its own at T0 and T60, but that the *13a-stop* mutation produced a small crawling speed reduction (reaching statistical significance at T60 only, Fig 3D). In contrast, the combined mutations significantly reduced the animals’ speed at both T0 and T60, although not to the same extent as in *tax-6(gf)*. Together, these data are consistent with our supposition that truncation of TAX-6 after residue 442 would create a deregulated, overactive protein, even if the phenotypes were systematically less severe in comparison to the shorter protein encoded by the *tax-6(gf)* allele. It is possible that the TAX-6(S443*) mutant protein retains some level of regulation as they keep the entire CBD, whereas this domain is partially truncated in TAX-6(GF), which would explain the quantitative differences. Furthermore, our data show that isoform-specific gain-of-function mutations produce different phenotypic outcomes: on the one hand, TAX-6a isoform truncation is sufficient to inhibit thermo-nociceptive adaptation, and TAX-6b/c isoform truncation has no effect. On the other hand, TAX-6a isoform truncation on its own produces little effect on animal speed, and will require the concomitant truncation of TAX-6b/c to produce a more salient effect. We considered two hypothetical models explaining this latter observation. In the first model, Calcineurin signaling up-regulation in multiple tissues would act synergistically to impair locomotion. In the second model, locomotion impairment would actually need the expression of the two truncated TAX-6 variants in reason of protein sequence divergence between the two isoforms. Because truncated TAX-6a and truncated TAX-6b/c proteins only differ in their penultimate residue (REE* in TAX-6a, versus RHE* in TAX-6b/c) the second model seemed less likely. We nevertheless devised an experiment to reject the second model. We generated a line combining the *13a-stop* and the *Δ*13bc** mutations and which should ubiquitously express truncated TAX-6a(S443*) in every *tax-6-*expressing tissue (Fig 4A and B). This line displayed strong phenotypes, with elevated naïve heat-evoked reversal rate similar to that in *tax-6(gf)* (Fig 4C, T0), impaired thermo-nociceptive adaptation (Fig 4C, T60) similar to that in *13a-stop* single mutants and speed reduced to levels significantly lower than in the *13a-stop* single mutant or wild type (Fig 4D). These results indicate that the truncated TAX-6a(S443*) mutant protein can produce a gain-of-function effect to modulate speed on its own, provided it reaches a broad-enough range of tissues and support our first model.

In summary, isoform-specific truncation mutation analysis in Fig 3 and 4 showed (i) that the truncation of TAX-6a is sufficient to inhibit thermo-nociceptive adaptation, but (ii) that the truncation of the two isoforms is required to constitutively reduce animal speed. The simplest interpretation of these results would be that TAX-6 signaling overactivation acts via the nervous system to control heat-evoked reversal, whereas it controls crawling speed via a larger set of tissues. These latter could include neurons and muscle, as most likely place of action to control locomotion.

### Overactivation of Calcineurin/TAX-6 in muscle and neurons synergize to reduce animal speed

**Fig 5:**
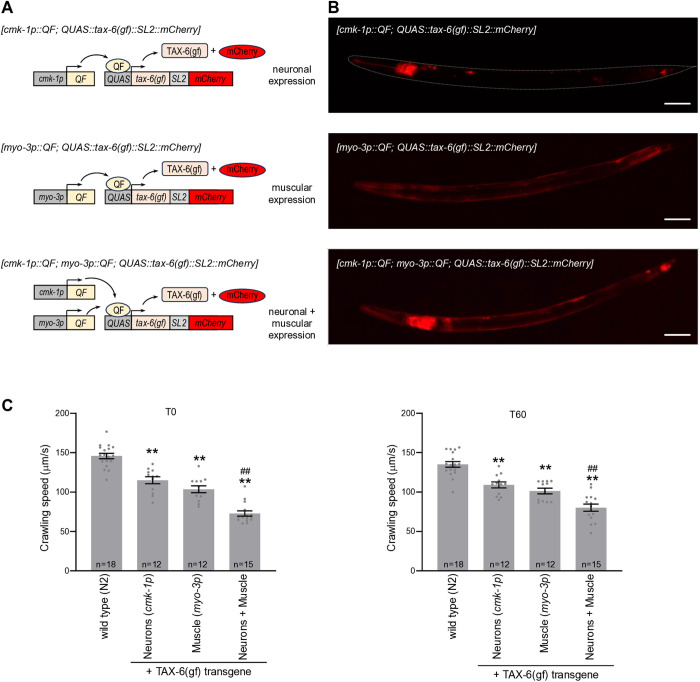
Calcineurin signaling up-regulation in muscle and neurons synergize to reduce crawling speed. **(A)** Schematic of the Q-system-based transgenes use for the tissue-specific expression of TAX-6(GF) overactive protein. *[cmk-1p::QF]* and *[myo-3p::QF]* transgenes lead to the tissue-specific expression of the transcriptional activator QF in neurons and muscle, respectively. A second transgene, *[QUAS::tax-6(gf)::SL2::mCherry]*, is then trans-activated by QF, leading to the production of two proteins: the over-active TAX-6(GF) and a mCherry cytoplasmic co-marker. **(B)** Representative epifluorescence microscopy images of the corresponding lines showing mCherry reporter expression in the nervous system, body wall muscle and both tissues, respectively. Scale bar: 100 μm. **(C)** Crawling speed measured in the absence of any heat stimulation. Each point corresponds to one assay scoring at least 50 animals. Average (grey bars) and s.e.m. (error bars) with indicated n representing the number of independent assays. T0: scoring of naïve animals; T60: scoring of animals exposed to repeated heat stimulation for 60 min. **, *p* < .01 versus N2(WT); ##, *p* < .01 versus each single transgene by Bonferroni-Holm post-hoc tests.

To further test the above-outlined model, in which TAX-6 would operate from both neurons and muscle to control locomotion, we created Q-system-based transgenic lines [33] over-expressing TAX-6(GF) truncated protein (with a sequence matching that found in *tax-6(gf)*) in a wild type background (Fig 5A). In separate lines, we used the *cmk-1p* promoter for expression in neurons, the *myo-3p* promoter for expression in body wall muscles, and a combination of the two promoters to simultaneously target both tissues (Fig 5B). The transgenes included a SL2::mCherry sequence enabling us controlling for proper tissular targeting (Fig 5C). We found that neuronal over-activation of TAX-6 in neurons or muscles caused a significant reduction in crawling speed at both T0 and T60 (Fig 5C). Moreover, the joint over-activation of TAX-6 signaling in both tissues caused a further decrease under both conditions. The cumulative effect supports a model in which TAX-6 signaling independently control locomotion from muscle and neurons, respectively, which is in line with the observations made with isoform-specific genetic manipulations indicating the need for gain-of-function mutations affecting both tissues in order to impair speed (Fig 3 and 4).

### Combined S443A phosphosite mutations in both TAX-6a and TAX-6b/c down-regulate locomotion but have no impact on heat-evoked reversals

**Fig 6:**
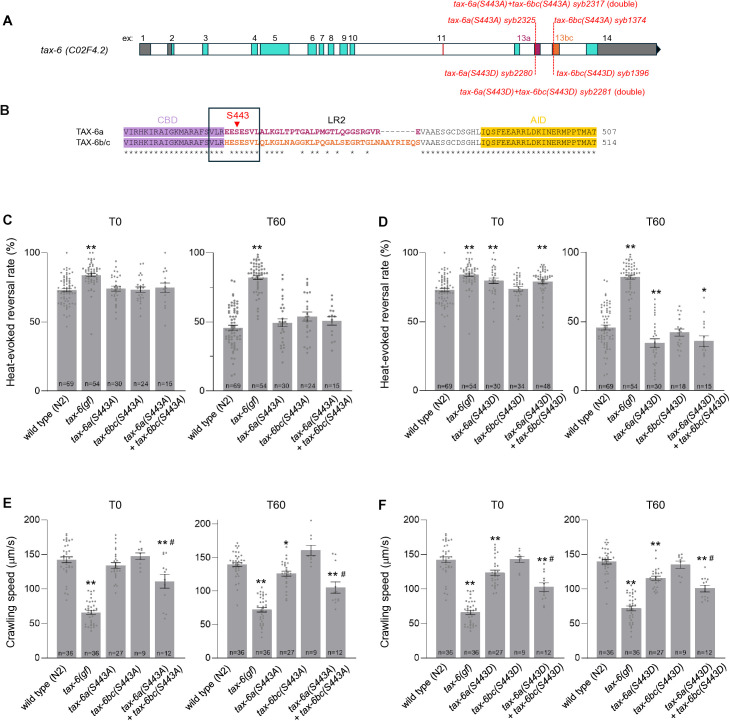
Isoform-specific impact of TAX-6 S443 phosphosite mutations. **(A)** Schematic representation of the *tax-6* genomic locus with description of the S443A (non-phosphorylatable) and S443D (phospho-mimetic) mutation-causing alleles: *tax-6a(S443A) syb2325*, *tax-6bc(S443A) syb1374*, *tax-6a(S443A)+tax-6bc(S443A) syb2317* double mutant, *tax-6a(S443D) syb2280*, *tax-6bc(S443D) syb1396*, and *tax-6a(S443D)+tax-6bc(S443D) syb2281* double mutant*.* **(B)** TAX-6 variant protein sequences showing the Linker Region 2 (LR2) connecting the Calmodulin binding domain (CBD) and the Auto-inhibitory domain (AID). S443 (in red) is present in both TAX-6a and TAX-6b/c isoforms as part of similar CMK-1 substrate recognition consensus (boxed). **(C-D)** Heat-evoked reversal scored in the indicated genotypes. Results as fraction of reversing animals. Each point corresponds to one assay scoring at least 50 animals. Average (grey bars) and s.e.m. (error bars) with indicated n representing the number of independent assays. T0: scoring of naïve animals; T60: scoring of animals exposed to repeated heat stimulation for 60 min. **(E-F)** Crawling speed measured in the absence of any heat stimulation and reported as under C-D. **, *p* < .01, * *p* < .05 versus N2(WT); #, *p* < .05 versus corresponding *tax-6a* single mutant by Bonferroni-Holm post-hoc tests. Wild type and *tax-6(gf)* data are replicated from Fig 3.

Calcineurin-dependent regulation of thermal nociception was previously shown to involve multiple antagonistic interactions with the Calcium/calmodulin-dependent protein kinase-1 (CMK-1) [28]. Interestingly, CMK-1 can phosphorylate Ser443 of TAX-6 *in vitro*. Ser443 is located in the LR2 region and, in *C. elegans*, is encoded in both exon 13a and exon 13bc in an homologous sequence context (Fig 6A and B) [28]. In particular, this serine is part of an VLREE**S**ESVL motif in TAX-6a and VLRHE**S**ESVL motif in the TAX-6b/c, both of which follow the ΦxRxx(S/T)xxxΦ substrate recognition consensus of CMK-1 (where Φ represents hydrophobic residues).

In order to assess the contributions of S443 phosphorylation across TAX-6 isoforms, we started by generating a series of CRISPR-Cas9-edited lines substituting this serine with a non-phosphorylatable alanine residue. Missense mutations were introduced in exon 13a to create a TAX-6a(S443A) mutant, in exon 13bc to create a TAX-6b/c(S443A) mutant or in both exons to create a double mutant affecting both isoforms (Fig 6A). We observed no marked heat-evoked reversal phenotype at either T0 or T60 in animal carrying the S443A mutations alone or in combination (Fig 6C). Next, we examined the impact of S443A mutations on locomotion. In single mutants, we found that TAX-6b/c(S443A) produced no effect on speed at either T0 and T60 and that the TAX-6a(S443A) mutation produced only a small crawling speed reduction at T60 (Fig 6E). In contrast, the double mutant displayed a significant speed reduction at T0 and T60, an effect that was stronger than that in TAX-6a(S443A) single mutants at T60 (Fig 6E). The speed reduction was not as marked as compared to *tax-6(gf)* mutants.

Collectively, our observations with S to A mutations mimicking a constitutive dephosphorylation suggest that the phosphorylation of S443 is largely dispensable for the control thermo-nociceptive adaptation, but is required for normal locomotion, for which we noted an asymmetrical S443 requirement in the different isoforms. This asymmetry could ensue from the facts that (i) Calcineurin signaling acts in both neurons and muscle to control locomotion, (ii) the two tissues asymmetrically express the two isoforms (neurons expressing only TAX-6a and muscle expressing mainly TAX-6b/c but also TAX-6a at a lower level, Fig 1) and (iii) the two tissues also asymmetrically express CMK-1 (the kinase being solely expressed in neurons).

### A S443D phospho-mimetic mutation in TAX-6a increases the responsiveness in naïve animals, but enhances the adaptation effect caused by repeated stimuli

Next, we examined the impact of S443D mutations (Fig 6A), which are expected to mimic permanent phosphorylation of S443. TAX-6b/c(S443D) single mutants produced normal heat-evoked reversals at T0 and T60. In contrast, TAX-6a(S443D) single mutants, as well as TAX-6a(S443D)+TAX-6b/c(S443D) double mutants behaved quite differently from wild type. At T0 they displayed elevated responses, thus resembling *tax-6(gf)* mutants (Fig 6D). At T60, they displayed enhanced thermo-nociceptive adaptation, manifesting as a more pronounced reduction in heat-evoked reversals as compared to wild type, and representing the opposite phenotype as compared to *tax-6(gf)* mutants (Fig 6D). To examine in more details the adaptation kinetics in these mutants, we acquired an intermediate timepoint after 30 min of repeated heat stimulations (T30, S5A Fig). Results indicated an accelerated adaptation in TAX-6a(S443D) mutants and in TAX-6a(S443D)+TAX-6b/c(S443D) double mutants, but, again, no effect in TAX-6b/c(S443D) mutants.

**Fig 7:**
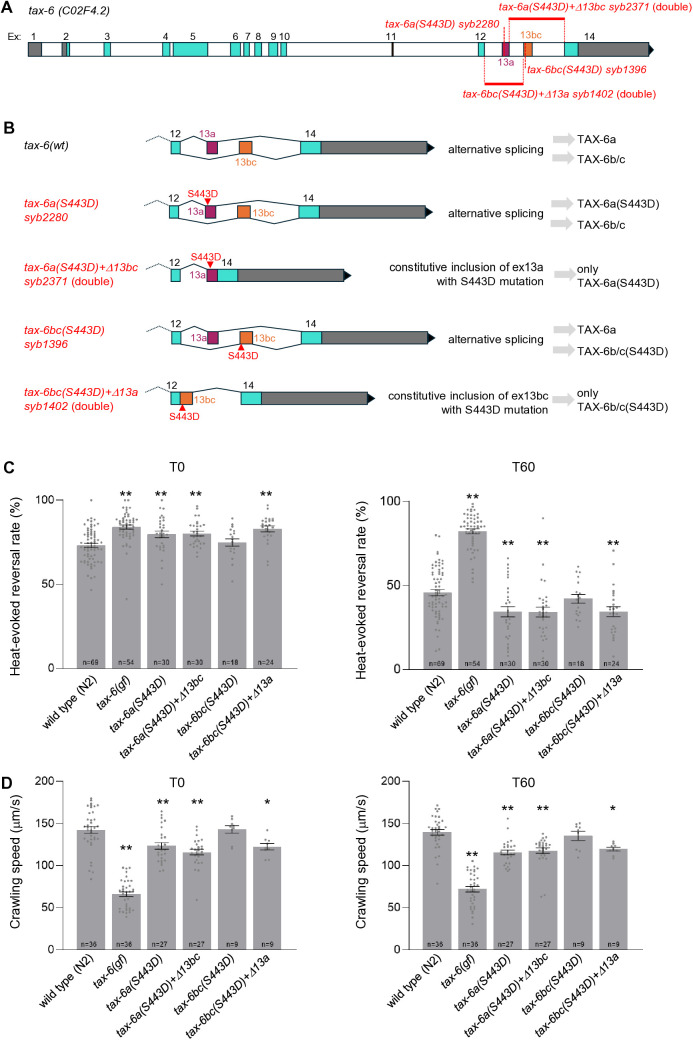
TAX-6b/c(S443D) phospho-mimetic mutations impact behavior when TAX-6a is genetically ablated. **(A)** Schematic representation of the *tax-6* genomic locus with description of the S443D (phospho-mimetic) single mutation-causing alleles affecting either isoform (*tax-6a(S443D) syb2280*, *tax-6bc(S443D) syb1396*), and of the alleles where the alternative isoform is concomitantly deleted (*tax-6a(S443D)+Δ13bc syb2371*, *tax-6bc(S443D)+Δ13a syb1402*). **(B)** Predicted impact of mutation on spliced transcripts and TAX-6 variant expression in these mutants. **(C)** Heat-evoked reversal scored in the indicated genotypes. Results as fraction of reversing animals. Each point corresponds to one assay scoring at least 50 animals. Average (grey bars) and s.e.m. (error bars) with indicated n representing the number of independent assays. T0: scoring of naïve animals; T60: scoring of animals exposed to repeated heat stimulation for 60 min. **(D)** Crawling speed measured in the absence of any heat stimulation and reported as under **C.** **, *p* < .01, *, *p* < .05 versus N2(WT) by Bonferroni-Holm post-hoc tests. Wild type and *tax-6(gf)* data are replicated from Fig 3.

Together these data with mutations mimicking a constitutive phosphorylation suggests that S443 phosphorylation in TAX-6a, but not in TAX-6b/c, is sufficient to elevate the responsiveness of naïve animals and to promote thermo-nociceptive adaptation. Is this isoform-specific effect due to differential isoform expression across tissues or to the actual difference in protein sequence in the C-term portion of the respective isoforms? To address this question, we combined each of the S443D mutation with a deletion mutation removing the other alternative isoforms (Fig 7A and B). The *tax-6a(S443D)* mutation was combined with the *Δ*ex13bc** deletion, to create a dual mutant that will ubiquitously express the phospho-mimetic TAX-6a(S443D) mutant protein in every *tax-6*-expressing tissues. In a separate line, the *tax-6bc(S443D)* was combined with the *Δ*ex13a** deletion, to create a dual mutant that will ubiquitously express the phospho-mimetic TAX-6b/c(S443D) mutant protein in every *tax-6*-expressing tissues. We found that both lines behaved like TAX-6a(S443D), showing up-regulated heat-evoked reversals at T0 as well as enhanced adaptation at T30 and T60, and thus behaving differently from TAX-6b/c(S443D) single (Fig 7C and S5B Fig)). We conclude that having a phospho-mimetic mutation in TAX-6 can modulate heat-evoked reversals regardless of C-terminal protein sequence variations across isoforms, as long as the phospho-mimetic TAX-6 mutant protein is expressed in *tax-6a*-expressing tissues. Therefore, the neuronal expression pattern of TAX-6a is probably much more relevant to the isoform-specific noxious heat avoidance phenotype, than the divergent protein sequence in the context of phospho-mimetic mutations.

### A S443D phospho-mimetic mutation in TAX-6a down-regulates locomotion

Next, we examined the impact of S443D mutations on crawling speed. The single *tax-6b/c(S443D)* mutation did not alter locomotion at T0 and T60 (Fig 6F). In contrast, both *tax-6a(S443D)* and *tax-6a(S443D)+tax-6b/c(S443D)* double mutants crawled slower than wild type at T0 and T60 (Fig 6F). These results suggest that the constitutive phosphorylation of TAX-6b/c is not sufficient to reduction locomotion speed, but that the constitutive phosphorylation of TAX-6a is and that the effect can be slightly enhanced when TAX-6b/c is concomitantly affected by the phospho-mimetic mutation on S443. Next, to determine if the differential effect of TAX-6(S443D) mutations across isoforms was due to sequence differences or expression pattern differences, we examined the speed of dual mutants combining alternative exon deletions and S443D mutations (Fig 7A). We found that both *tax-6a(S443D)+Δ*13bc** and *tax-6bc(S443D)+Δ*13a** lines behaved like *tax-6a(S443D)*, showing decreased speed (Fig 7D). We conclude that S443 phospho-mimetic mutations in TAX-6 can modulate speed regardless of C-terminal protein sequence variations across isoforms, as long as the phospho-mimetic TAX-6 mutant protein is expressed in *tax-6a*-expressing tissues.

### A model of TAX-6 variant-specific functions and phosphorylation-dependent modulation across tissues

It is quite remarkable that the residue corresponding to worm TAX-6 S443, as well as the surrounding CaMK substrate consensus sequence, are conserved across alternative CnA isoforms in worm, fish and mammals (S1 Fig.). This is a first indirect indication for an important function. In addition, the results of our S443A and S443D mutant analysis provide direct evidence that S443 is indeed important for the proper regulation of TAX-6 in *C. elegans*. Whereas we cannot rule out a broader structural alteration caused by S443 mutations, the notable phenotypic divergence between S443A and S443D mutants regarding noxious heat-avoidance suggests that phosphorylation-based regulation is involved.

An important note of caution should be made regarding the interpretation of results from mutants expressing TAX-6b/c(S443D). While the phosphorylated form of TAX-6a is expected to be encountered in the nervous system of wild type animals when CMK-1 is active, this kinase is not expressed in muscle and therefore cannot phosphorylate TAX-6b/c. Even if another unknown kinase could potentially take over this function in muscle, it is possible that TAX-6b/c is simply never phosphorylated in muscle and that the (globally minor) impact of TAX-6b/c S443D mutation reflects a purely artificial situation. Overall, S443A and S443D mutations affecting TAX-6a produced stronger phenotypic effects, which can be attributed to altered Calcineurin signaling in neurons, where S443 is most likely subject to phosphorylation-based regulation.

**Fig 8:**
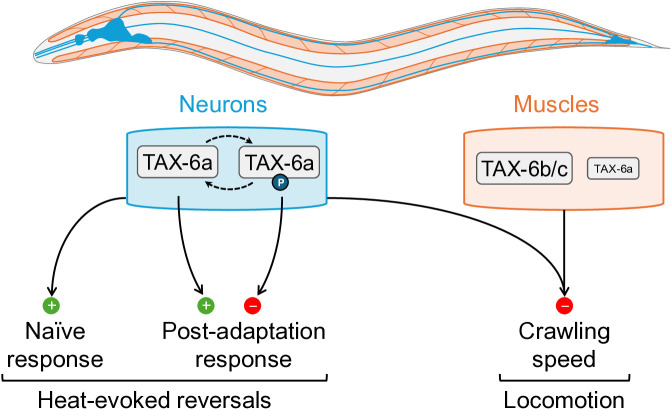
A model of TAX-6 variant-specific functions and phosphorylation-dependent modulation across tissues. The model graphically summarizes the main conclusions of the present study regarding the expression and function of splice variants that diversify the LR2 region in the regulatory domain of TAX-6/CnA in *C. elegans*. TAX-6a is the predominant isoform in neurons, whereas muscle is enriched in TAX-6b/c, with a lower level of TAX-6a. Phosphorylation of S443 is most likely tacking place to modulate TAX-6a function in neurons. TAX-6b/c and TAX-6a in muscle are shown in an unphosphorylated state only. The reason is that we have no indication that S443 phosphorylation would take place in a tissue that does not express CMK-1, but one should keep in mind that it could still be achieved by an unknown alternative kinase. TAX-6a signaling in neurons produces mostly a heat-evoked reversal promoting effect, both in naïve animals (T0) and in post-adaptation animals submitted to one-hour of repeated stimulations (T60). The latter effect at T60 is reverted by S443 phosphorylation, which implies that the TAX-6-mediated regulation of this experience-dependent behavioral trait is selectively regulated by phosphorylation, whereas the constitutive response in naïve animals is not. Unlike reversal responses that are regulated by Calcineurin signaling primarily in the nervous system, Calcineurin-mediated locomotion regulation involves the synergistic action of TAX-6a in neurons and of TAX-6b/c in muscle.

At this stage, we cannot categorize the S443 phosphosite mutants as either gain- or loss-of-functions, since their phenotypes qualitatively and quantitatively diverge from our two *tax-6(lf)* and *tax-6(gf)* reference lines. Instead, we propose that S443A/D mutants constitute a novel class of mutants with impaired regulation. The *enhanced adaptation* phenotype of TAX-6a(S443D) mutants suggest that TAX-6a(S443) dephosphorylation in neurons is part of a process that normally counteract adaptation upon repeated stimulations. Furthermore, it is interesting that the TAX-6a(S443D) phenotype (Fig 6C) resembles that of *Δ*13a** mutants (Fig 2C), who express only TAX-6b/c in neurons (whereas neurons should normally almost exclusively express TAX-6a). A simple hypothetical explanation of this phenotypic convergence would be that the S443 dephosphorylation, which is essential in the nervous system to inhibit adaptation, could only be properly achieved on TAX-6a, but not on TAX-6b/c when mis-expressed in the nervous system. Future studies comparing the biochemical properties, subcellular localization and interacting partners of TAX-6 isoforms will be needed to fully elucidate the underlying mechanism. Nevertheless, the present set of *in vivo* data allowed us sketching a graphical model summarizing the places of action and phosphorylation-dependent regulation of TAX-6 variants in controlling locomotion and heat-evoked reversal (Fig 8). Our data suggest a marked role for phosphorylation-dependent regulation in neurons (notably known to express CMK-1), but little role in muscles (which do not express CMK-1). Therefore, we depicted TAX-6a in neurons as either phosphorylated or dephosphorylated and TAX-6 in muscle without any phosphorylation (Fig 8). Key features of the model are, first, that active TAX-6 signaling in both neurons and muscle can synergize to reduce locomotion. Second, that active TAX-6a signaling in neurons globally tends to up-regulate heat-evoked reversal response in naïve animals and following repeated stimulations, hence inhibiting adaptation. And third, that TAX-6a signaling in neurons is further modulated by phosphorylation on S443, with constitutive phosphorylation reinforcing naïve responsiveness, but reverting the impact of calcineurin signaling to down-regulate heat-evoked reversals and enhance adaptation. In summary, our model presents the non-redundant functions of TAX-6 isoforms working in a tissue-specific manner to control both constitutive and experience-dependent behaviors.

## Conclusion and perspective

Our study has revealed that the LR2 region, which links the CBD to the AID domain in canonical Calcineurin A catalytic subunits, represents a sequence variation hotspot with different forms of alternative splicing diversifying the encoded isoforms across vertebrates and invertebrates. Follow-up functional studies using genome engineering to create isoform-specific mutations in *C. elegans* have confirmed the isoform-specific contributions to the regulation of distinct behavioral traits shaped by Calcineurin signaling, as well as the tissue-specific relevance of isoform-specific phosphorylation events. In short, our data support a general model in which Calcineurin signaling (i) controls noxious-heat-evoked reversals in TAX-6a-expressing neurons, and (ii) modulates speed in both TAX-6a and TAX-6b/c-expressing tissues, including neurons and muscles. Additionally, phosphorylation-dependent regulation of TAX-6 plays a significant role in the nervous system to modulate the impact of Calcineurin signaling during experience-dependent behavioral plasticity. Since the role of the LR2 region in CnA protein function is not well defined, the functional consequences of LR2 variation remain speculative. This region could potentially influence several aspects of CnA activity, including its catalytic function, binding to CaM or the regulatory subunit CnB, or interactions with other proteins that guide the phosphatase toward specific substrates.

The functional significance of LR2 diversification through alternative splicing in other species also remains largely unexplored. However, given the high evolutionary conservation of this alternative splicing hotspot, we anticipate that they will also be relevant in other species. Overall, our study provides the first proof of the importance of alternative splicing in this region in controlling behavioral functions via both neuronal and non-neuronal tissues. Our findings lay the foundation for further research, including in humans, where similar splicing events diversify all three CnA paralogues, which may be important for understanding the many biological functions and dysfunctions associated with Calcineurin activity in various tissues.

## Methods

### Worm strains, maintenance and synchronization

Nematode strains were grown on nematode growth media (NGM) plates seeded with *E. coli* OP50 bacteria at 20 °C. All *C. elegans* strains used in this study are listed in S1 File. Genome engineering was performed by SunyBiotech (Fuzhou, China). Resulting lines were back-crossed twice with wild type (N2) prior to analysis. The genomic sequences of new *tax-6* alleles generated for the present study are included in S1 File. Sanger sequencing of the full genomic region surrounding the CRISPR-edited loci was performed to confirm the presence of the intended mutations.

### Molecular cloning and transgenesis

LR recombination reactions (Gateway LR Clonase, Invitrogen) were used to create expression plasmids according to the manufacturer’s instructions using previously described entry clones [34–36]. All constructed plasmids were transformed into DH5a competent *E. coli* (NEB C2987H) and purified with the GenElute HP Plasmid miniprep kit (Sigma). DNA constructs were microinjected together with the *[unc-122p::GFP]* co-injection marker (each at a concentration of 20 ng/μl) in the worm gonad according to a standard protocol [37]. Details of plasmids and transgenic lines are presented in S1 File.

### Fluorescence imaging

Imaging of mCherry co-markers for *tax-6(gf)* expression in neurons (*cmk-1p*) and muscles (*myo-3p*) was performed in sodium azide-treated animals using a Zeiss Axioplan2 fluorescence microscope as previously described [38,39].

### Worm preparation for behavior recordings

For animal synchronization, gravid adult worms were treated with hypochlorite solution according to standard protocols. Isolated embryos were rinsed twice with sterile water and once with M9 buffer. Embryos were resuspended in M9 buffer, plated on OP-50 seeded NGM plates (200–300 embryos/plate) and incubated at 20 °C until the very beginning of egg laying (~65 h). For experiments with stable extrachromosomal array-containing transgenic lines, fluorescently labelled worms were picked and placed on new NGM plates 16–19 h prior to experiment to allow recovery.

The day of experiment, worms were washed off the plates with distilled water, placed into 1.5 ml tubes, washed twice more to eliminate bacterial remnants and placed on unseeded NGM plates, which had been left in a laminar flow hood for 3 h prior to the experiment to ensure dry surface. Washed worms were left to disperse and acclimate in the experimental room for 60 min, with the last 3 min spent with the lid open.

### Heat-evoked reversal and crawling speed measures

Behavioral data acquisition was performed on plates with open lids as previously described [28]. Briefly, for heat stimulation and behavior recording, the INFERNO system [31] was used. The heat stimulation program was applied at T0 (naïve animals) and T60 (adapted animals). The program consisted in a 40 s baseline period without any heat stimulation and 4 s with 400 W heating (4 infra-red lamps turned on). Movies were recorded using a DMK 33U × 250 camera mounted on a macro zoom with the IC capture software (The Imaging Source), at 8 frames per second, at a 1600 × 1800pixel resolution, and the resulting.AVI file was encoded as Y800 8-bit monochrome. For the adaptation worm plates (T60) were placed under the ThermINATOR system for 1h providing infinitely looping temperature program composed of 4 s of IR lamps stimulation, followed by 20 s ISI with the lamps turned off. For movie analysis, we used the Multi-Worm Tracker 1.3.0 (MWT) [40] with configuration settings previously described [31]. A previously described Python script [31] was used to flag the frame of reversal occurrence and extract speed data. Each reported data point corresponds to the results of one assay plate, scoring at least 50 animals. Crawling speed average was calculated using the 40-s baseline before the heat pulse.

### Number of experiments and statistical analysis

For the behavioral analysis of each strain, a minimum of 3 replicates were performed on 3 separate days, running wild-type N2 strain as a control. Actual numbers of replicates per condition are indicated directly in the different figures, where *n* represents the number of separate assays, each scoring more than 50 animals. For experiments with transgenic animals (data in Fig 5), we tested three independent transgenic lines and, because their scores were similar, reported results as aggregates. Jamovi was used for conduction of ANOVAs (The jamovi project (2022), jamovi (Version 2.3) [Computer Software]; obtained from (https://www.jamovi.org). Post hoc tests were used to compare each of the mutants with wild type (N2) or *tax-6(gf)* using Bonferroni–Holm correction. All *p-*values are reported in S2 File.

## Supporting information

S1 Fig Conservation of alternative splicing in the LR2 of Calcineurin A. Protein sequence alignment showing the LR2 region and nearby Calmodulin binding domain (CBD) and auto-inhibitory domain (AID) (A) Comparison of *C. elegans* (*C.ele)* TAX-6a and TAX-6b/c with one mouse variant and the three paralogous human variants. (B) Comparison between *C. elegans* and the Zebrafish *Danio rerio (D. rer).* Accession: Q08209-2|PP2BA_HUMAN; P16298-3|PP2BB_HUMAN; P48454-2|PP2BC_HUMAN; P63328-2|PP2BA_MOUSE; Q08209|PP2BA_HUMAN; P16298|PP2BB_HUMAN; P48454|PP2BC_HUMAN; P63328|PP2BA_MOUSE.
(PDF)

S2 Fig *tax-6* gene models showing conservation of alternative splicing across nematode species in the region corresponding to *C. elegans* exon 13. (A) Gene structures of the *tax-6* gene orthologs of the indicated species were assembled from gene models and splice data in wormbase version WS292. The representations were arbitrarily aligned around alternative exon 13 of *C. elegans*. The genes are to scale. Actual size of the gene section depicted: *Caenorhabditis elegans*: 8731 bp; *Caenorhabditis briggsae*: 9786 bp; *Caenorhabditis brennerii*: 8378 bp*. Pristionchus pacificus*: 11724 bp*.* Boxes: exons, with known UTR as thinner boxes. Grey: constitutive exons. Color: alternative exons. (B) Genomic sequence alignment of alternative exon 13a and 13bc across the indicated nematode species. 10 bases of flanking intronic sequence are included. (C) Sequence alignment of the protein domains encoded by alternative exon 13a (top) and 13bc (bottom) in the indicated nematode species. Grey shades cover residues shared by the two alternative protein domains and strictly conserved across the four species.
(PDF)

S3 Fig *tax-6* isoform expression in individual neuron types (A) *tax-6* gene description with isoform definition as in Fig 1A (replicated panel). (B) Number of transcripts per million (TPM) measured for individual neuron types in the CeNGEN analysis. Generated on October 10, 2024 https://cengen.shinyapps.io/isoform_compare/
(PDF)

S4 Fig Altered noxious heat avoidance and locomotion in *tax-6* loss- and gain-of-function mutants. (A) Schematic representation of the *tax-6* genomic locus with description of the *tax-6(p675)* loss-of-function (lf) and *tax-6(jh107)* gain-of-function (gf) alleles. (B) Heat-evoked reversal scored in the indicated genotypes. Results as fraction of reversing animals. Each point corresponds to one assay scoring at least 50 animals. Average (grey bars) and s.e.m. (error bars) with indicated n representing the number of independent assays. T0: scoring of naïve animals. T60: scoring of animals exposed to repeated heat stimulation for 60 min. (C) Crawling speed measured in the absence of any heat stimulation and reported as under B. **, *p* < .01 versus N2(WT) control; ##, *p* < .01 versus *tax-6(gf)* by Bonferroni-Holm post-hoc tests.
(PDF)

S5 Fig Accelerated adaptation to repeated stimuli caused by *tax-6a(S443D)* mutation and by the *tax-6bc(S443D)* mutation in the absence of *tax-6a.* (A-B) The experiments were similar to those presented in Fig 6C and 7D, but the adaptation effect was assessed at an earlier timepoint (T30 here, versus T60 in the main figures). Heat-evoked reversal scored in the indicated genotypes. Results as fraction of reversing animals. Each point corresponds to one assay scoring at least 50 animals. Average (grey bars) and s.e.m. (error bars) with indicated n representing the number of independent assays. T0: scoring of naïve animals; T30: scoring of animals exposed to repeated heat stimulation for 30 min. **, *p* < .01 versus N2(WT) by Bonferroni-Holm post-hoc tests.
(PDF)

S1 File Strain list, plasmid list and sequences of engineered *tax-6* alleles. (DOCX)

S2 File Raw data used in figures. (XLSX)
