# Supplementary material for: Conserved Calcineurin A splice variants regulate both constitutive and experience-dependent behaviors through tissue-specific signaling
Source: PLoS Genet. 2025 Sep 26;21(9):e1011884. doi: 10.1371/journal.pgen.1011884 (PMC12503234; doi:10.1371/journal.pgen.1011884)
Supplement: S1 Fig — Protein sequence alignment showing the LR2 region and nearby Calmodulin binding domain (CBD) and auto-inhibitory domain (AID) (A) Comparison of C. elegans (C.ele) TAX-6a and TAX-6b/c with one mouse variant and the three paralogous human variants. (B) Comparison between C. elegans and the Zebrafish Danio rerio (D. rer). Accession: Q08209-2|PP2BA_HUMAN; P16298-3|PP2BB_HUMAN; P48454-2|PP2BC_HUMAN; P63328-2|PP2BA_MOUSE; Q08209|PP2BA_HUMAN; P16298|PP2BB_HUMAN; P48454|PP2BC_HUMAN; P63328|PP2BA_MOUSE. (PDF) [file pgen.1011884.s001.pdf]

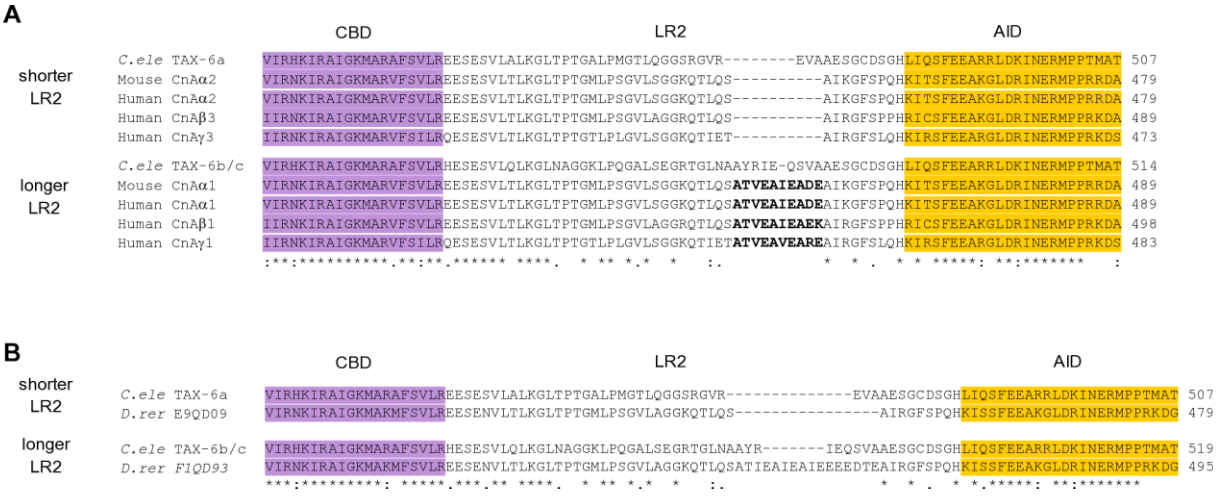

S1 Fig. Conservation of alternative splicing in the LR2 of Calcineurin A.

Protein sequence alignment showing the LR2 region and nearby Calmodulin binding domain (CBD) and auto-inhibitory domain (AID) (A) Comparison of *C. elegans* (*C.ele)* TAX-6a and TAX-6b/c with one mouse variant and the three paralogous human variants. (B) Comparison between *C. elegans* and the Zebrafish *Danio rerio (D. rer).* Accession: Q08209-2|PP2BA_HUMAN; P16298-3|PP2BB_HUMAN; P48454-2|PP2BC_HUMAN; P63328-2|PP2BA_MOUSE; Q08209|PP2BA_HUMAN; P16298|PP2BB_HUMAN; P48454|PP2BC_HUMAN; P63328|PP2BA_MOUSE
