# Supplementary material for: Conserved Calcineurin A splice variants regulate both constitutive and experience-dependent behaviors through tissue-specific signaling
Source: PLoS Genet. 2025 Sep 26;21(9):e1011884. doi: 10.1371/journal.pgen.1011884 (PMC12503234; doi:10.1371/journal.pgen.1011884)
Supplement: S2 Fig — (A) Gene structures of the tax-6 gene orthologs of the indicated species were assembled from gene models and splice data in wormbase version WS292. The representations were arbitrarily aligned around alternative exon 13 of C. elegans. The genes are to scale. Actual size of the gene section depicted: Caenorhabditis elegans: 8731 bp; Caenorhabditis briggsae: 9786 bp; Caenorhabditis brennerii: 8378 bp. Pristionchus pacificus: 11724 bp. Boxes: exons, with known UTR as thinner boxes. Grey: constitutive exons. Color: alternative exons. (B) Genomic sequence alignment of alternative exon 13a and 13bc across the indicated nematode species. 10 bases of flanking intronic sequence are included. (C) Sequence alignment of the protein domains encoded by alternative exon 13a (top) and 13bc (bottom) in the indicated nematode species. Grey shades cover residues shared by the two alternative protein domains and strictly conserved across the four species. (PDF) [file pgen.1011884.s002.pdf]

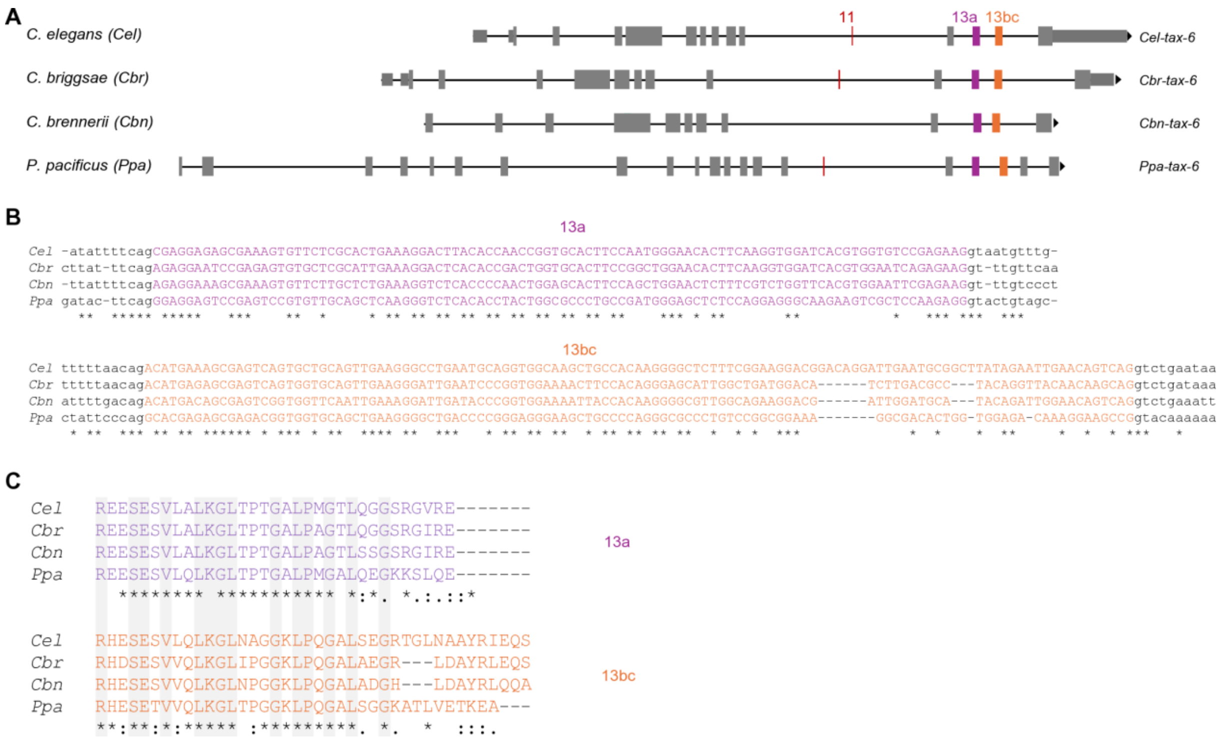

S2 Fig. *tax-6* gene models showing conservation of alternative splicing across nematode species in the region corresponding to *C. elegans* exon 13.

(A) Gene structures of the *tax-6* gene orthologs of the indicated species were assembled from gene models and splice data in wormbase version WS292. The representations were arbitrarily aligned around alternative exon 13 of *C. elegans*. The genes are to scale. Actual size of the gene section depicted: *Caenorhabditis elegans*: 8731 bp; *Caenorhabditis briggsae*: 9786 bp; *Caenorhabditis brennerii*: 8378 bp. *Pristionchus pacificus*: 11724 bp. Boxes: exons, with known UTR as thinner boxes. Grey: constitutive exons. Color: alternative exons. (B) Genomic sequence alignment of alternative exon 13a and 13bc across the indicated nematode species. 10 bases of flanking intronic sequence are included. (C) Sequence alignment of the protein domains encoded by alternative exon 13a (top) and 13bc (bottom) in the indicated nematode species. Grey shades cover residues shared by the two alternative protein domains and strictly conserved across the four species.
