# Supplementary material for: Conserved Calcineurin A splice variants regulate both constitutive and experience-dependent behaviors through tissue-specific signaling
Source: PLoS Genet. 2025 Sep 26;21(9):e1011884. doi: 10.1371/journal.pgen.1011884 (PMC12503234; doi:10.1371/journal.pgen.1011884)
Supplement: S4 Fig — (A) Schematic representation of the tax-6 genomic locus with description of the tax-6(p675) loss-of-function (lf) and tax-6(jh107) gain-of-function (gf) alleles. (B) Heat-evoked reversal scored in the indicated genotypes. Results as fraction of reversing animals. Each point corresponds to one assay scoring at least 50 animals. Average (grey bars) and s.e.m. (error bars) with indicated n representing the number of independent assays. T0: scoring of naïve animals. T60: scoring of animals exposed to repeated heat stimulation for 60 min. (C) Crawling speed measured in the absence of any heat stimulation and reported as under B. **, p < .01 versus N2(WT) control; ##, p < .01 versus tax-6(gf) by Bonferroni-Holm post-hoc tests. (PDF) [file pgen.1011884.s004.pdf]

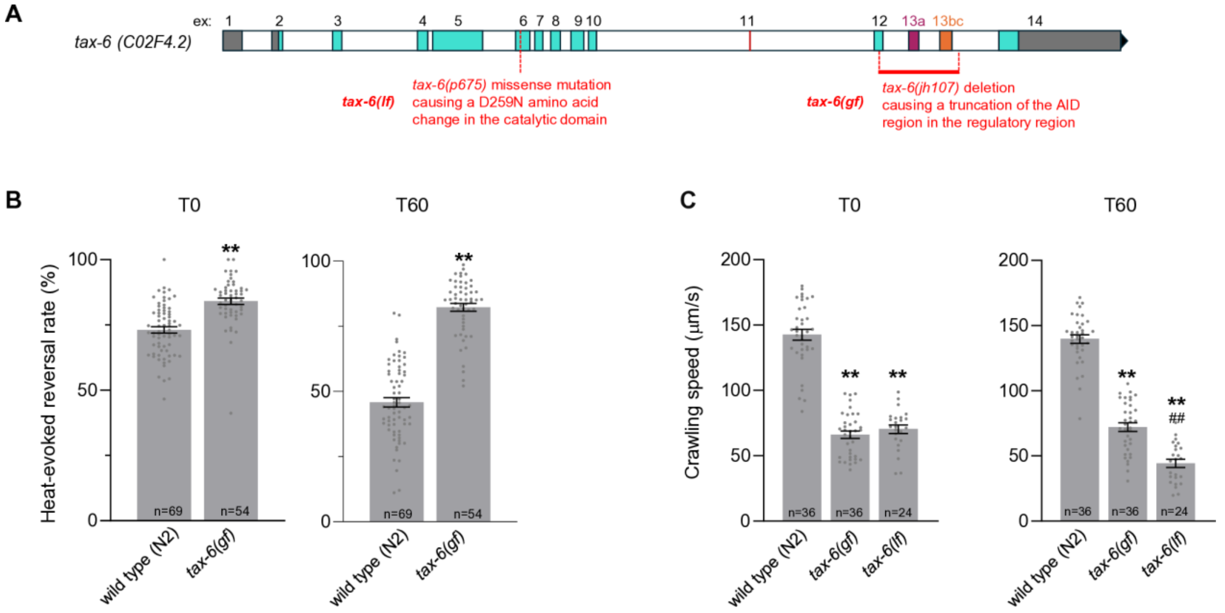

S4 Fig. Altered noxious heat avoidance and locomotion in *tax-6* loss- and gain-of-function mutants.

(A) Schematic representation of the *tax-6* genomic locus with description of the *tax-6(p675)* loss-of-function (lf) and *tax-6(jh107)* gain-of-function (gf) alleles. (B) Heat-evoked reversal scored in the indicated genotypes. Results as fraction of reversing animals. Each point corresponds to one assay scoring at least 50 animals. Average (grey bars) and s.e.m. (error bars) with indicated n representing the number of independent assays. T0: scoring of naïve animals. T60: scoring of animals exposed to repeated heat stimulation for 60 min. (C) Crawling speed measured in the absence of any heat stimulation and reported as under B. **, p<.01 versus N2(WT) control; ##, p<.01 versus *tax-6(gf)* by Bonferroni-Holm post-hoc tests.
