# Supplementary material for: Conserved Calcineurin A splice variants regulate both constitutive and experience-dependent behaviors through tissue-specific signaling
Source: PLoS Genet. 2025 Sep 26;21(9):e1011884. doi: 10.1371/journal.pgen.1011884 (PMC12503234; doi:10.1371/journal.pgen.1011884)
Supplement: S5 Fig — (A-B) The experiments were similar to those presented in Fig 6C and 7D, but the adaptation effect was assessed at an earlier timepoint (T30 here, versus T60 in the main figures). Heat-evoked reversal scored in the indicated genotypes. Results as fraction of reversing animals. Each point corresponds to one assay scoring at least 50 animals. Average (grey bars) and s.e.m. (error bars) with indicated n representing the number of independent assays. T0: scoring of naïve animals; T30: scoring of animals exposed to repeated heat stimulation for 30 min. **, p < .01 versus N2(WT) by Bonferroni-Holm post-hoc tests. (PDF) [file pgen.1011884.s005.pdf]

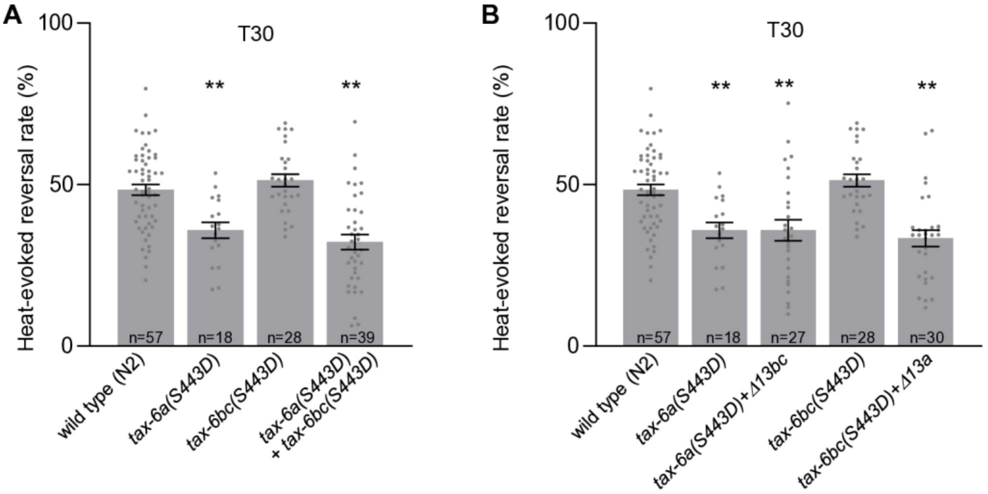

S5 Fig. Accelerated adaptation to repeated stimuli caused by *tax-6a(S443D)* mutation and by the *tax-6bc(S443D)* mutation in the absence of *tax-6a*.

(A-B) The experiments were similar to those presented in Fig 6C and 7D, but the adaptation effect was assessed at an earlier time point (T30 here, versus T60 in the main figures). Heat-evoked reversal scored in the indicated genotypes. Results as fraction of reversing animals. Each point corresponds to one assay scoring at least 50 animals. Average (grey bars) and s.e.m. (error bars) with indicated n representing the number of independent assays. T0: scoring of naïve animals; T30: scoring of animals exposed to repeated heat stimulation for 30 min. **, $p<.01$ versus N2(WT) by Bonferroni-Holm post-hoc tests.
