# Supplementary material for: Conserved Calcineurin A splice variants regulate both constitutive and experience-dependent behaviors through tissue-specific signaling
Source: PLoS Genet. 2025 Sep 26;21(9):e1011884. doi: 10.1371/journal.pgen.1011884 (PMC12503234; doi:10.1371/journal.pgen.1011884)
Supplement: S1 File — (DOCX) [file pgen.1011884.s006.docx]

**S1 File**

**Content:**

- **Strain list**
- **Plasmid list**
- **Genome editing mutation description**

**Strain list**

| **Strain Name** | **Genotype** | **Comments** |
| --- | --- | --- |
| N2 | Wild type | Wild type (WT) |
| KJ306 | *tax-6(jh107) IV* | Obtained from CGC. |
| PR675 | *tax-6(p675) IV* | Obtained from CGC. |
| DAG1018 | *tax-6(syb1371) (13bc-stop)* | Created for this study |
| DAG1019 | *tax-6(syb1374) (tax-6bc S443A)* | Created for this study |
| DAG1020 | *tax-6(syb1369) (Δex13bc)* | Created for this study |
| DAG1035 | *tax-6(syb1384) (Δex13a)* | Created for this study |
| DAG1037 | *tax-6(syb1396) (tax-6bc S443D)* | Created for this study |
| DAG1038 | *tax-6(syb1389) (13a-stop)* | Created for this study |
| DAG1039 | *tax-6(syb1402) (tax-6bc S443D + Δex13a)* | Created for this study |
| DAG1040 | *tax-6(syb1391) (tax-6bc S443A + Δex13a)* | Created for this study |
| DAG1144 | *tax-6(syb2325) (tax-6a S443A)* | Created for this study |
| DAG1145 | *tax-6(syb2280) (tax-6a S443D)* | Created for this study |
| DAG1146 | *tax-6(syb2324) (tax-6a S443A+ Δex13bc)* | Created for this study |
| DAG1147 | *tax-6(syb2317) (tax-6a S443A + tax-6bc S443A)* | Created for this study |
| DAG1165 | *tax-6(syb2371) (tax-6a S443D + Δex13bc)* | Created for this study |
| DAG1166 | *tax-6(syb2282) (13a-stop + Δex13bc)* | Created for this study |
| DAG1167 | *tax-6(syb2281) (tax-6a S443D + tax-6bc S443D)* | Created for this study |
| DAG1168 | *tax-6(syb2354) (13a-stop + 13bc-stop)* | Created for this study |
| DAG2023/24/25 | *domEx2023-25[cmk-1p::QF;QUAS::tax-6(gf)::SL2::mCherry; unc-122p::GFP]* | Created for this study |
| DAG2026/27/28 | *domEx2026-28[myo-3::QF;QUAS::tax-6(gf)::SL2::mCherry; unc-122p::GFP]* | Created for this study |
| DAG2029, DAG2033/34 | *domEx2029/2033-34[cmk-1p::QF; myo-3::QF; QUAS::tax-6(gf)::SL2::mCherry; unc-122p::GFP]* | Created for this study |

**Plasmid list**

| **Promoter plasmids (multi-site gateway slot 1)** | | |
| --- | --- | --- |
| mg268 | *cmk-1p* (slot1 Entry) | Created previously (Schild et. al 2014) |
| dg1110 | *myo-3p* (slot1 Entry) | Gift from Chantal Wicky (UniFR, Switzerland) |
|  |  |  |
| **Coding sequence plasmids (multi-site gateway slot 2)** | | |
| dg240 | *QF* (slot2 Entry) | Created previously (Schild et. al 2014) |
|  |  |  |
| **3’ UTR and tagging plasmids (multi-site gateway slot 3)** | | |
| mg277 | *SL2::mCherry::unc-54 3’UTR* (slot3 Entry) | Previously described (Schild et. al 2014) |
| mg211 | *unc-54 3’UTR* (slot3 Entry) | gift from Marc Hammarlund (Yale University, CT, USA) |
|  | | |
| **Expression clones** | | |
| dg1059 | *QUAS::tax-6(gf)::SL2::mCherry* | Created previously (Rudgalvyte et. al 2024) includes the coding sequence found in *tax-6(jh107)*, referred to as *tax-6(gf)* |
| dg1111 | *myo-3p::QF::unc-54UTR* | Created through a LR recombination reaction between dg1110, dg240, mg211, dg560 |
| dg372 | *cmk-1p::QF::unc-54UTR* | Created through a LR recombination reaction between mg268, dg240, mg211, dg560 |
|  |  |  |
|  |  |  |
| **Co-injection marker** | | |
| dg396 | *unc-122p::*GFP | gift from Piali Sengupta (Brandeis University, MA, USA); Addgene plasmid # 8937 |
|  |  |  |
| **DEST plasmid for vector construction** | | |
| dg560 | *pDEST R4-R3* | gift from Marc Hammarlund (Yale University, CT, USA) |

**Genome editing mutation description:**

***tax-6(syb1371) (13bc-stop)***

wild type sequence

ACATGAA**AGC**GAGTCA**GTG**CTGCAGTTGAAGGGCCTGAATGCAGGTGGCAAGCTGCCACAAGGGGCTCTTTCGGAAGGACGGACAGGATTGAATGCGGCTTATAGAATTGAACAGTCAG

mutant sequence (1 nonsense + 1 synonymous mutation)

ACATGAA**TAG**GAGTCA**GTA**CTGCAGTTGAAGGGCCTGAATGCAGGTGGCAAGCTGCCACAAGGGGCTCTTTCGGAAGGACGGACAGGATTGAATGCGGCTTATAGAATTGAACAGTCAG

***tax-6(syb1374) (tax-6bc S443A)***

wild type sequence

ACATGAA**AGC**GAGTCA**GTG**CTGCAGTTGAAGGGCCTGAATGCAGGTGGCAAGCTGCCACAAGGGGCTCTTTCGGAAGGACGGACAGGATTGAATGCGGCTTATAGAATTGAACAGTCAG

mutant sequence (1 missense + 1 synonymous mutation)

ACATGAA**GCA**GAGTCA**GTA**CTGCAGTTGAAGGGCCTGAATGCAGGTGGCAAGCTGCCACAAGGGGCTCTTTCGGAAGGACGGACAGGATTGAATGCGGCTTATAGAATTGAACAGTCAG

***tax-6(syb1369) (Δex13bc)***

wild type sequence

TCGACACAAGATTCGAGCAATTGGAAAGATGGCAAGAGCATTCTCAGTTCTTCGgtaagttgattaatttttttcaattttaaaccgaatttgtatgaatttaaatagaaagtttttttctattatagaatgagcattttttttagttaacttttatttatttttgataacatatatcttaatttaatgtttctataaaaaaacaacatacttaaagcatgattttgacaatttttgttaattaatcatttttgattatttttttttcaaagttcataactctttatgtggtttttcatattttcagCGAGGAGAGCGAAAGTGTTCTCGCACTGAAAGGACTTACACCAACCGGTGCACTTCCAATGGGAACACTTCAAGGTGGATCACGTGGTGTCCGAGAAGgtaatgtttgtcctgatgtctcttcgatcctgatccgtccaaaatcgtttaaagtccgtcaaaaagtgtgtgtacttttctctaatcgaaatgcaactatacttaccgagttaactttcgaatcaaatgatattttctacgaaaaaacaataattcaatagaaaaaaggaaagagaaaaacgtagtaccacgctttttaacagACATGAAAGCGAGTCAGTGCTGCAGTTGAAGGGCCTGAATGCAGGTGGCAAGCTGCCACAAGGGGCTCTTTCGGAAGGACGGACAGGATTGAATGCGGCTTATAGAATTGAACAGTCAGgtctgaataataaaaattatctaaattatctatggtctgcaaatgtttaaaacatctgattctacaaaagtatggtttttttaactgaaagaacattgcttttggggcaacaccgacgttttcgttttcgaaaatgtttttaattatttttcttttcaaaattacaactttaaagttgctaaaaaaaaacaaatttgattagacaaccaggaatgaagaaaaatctaagcatctgcattcgattttttttcttttttgtgacgagacccattcaaatagccacgtgcgactttaaagtattaatttcgaaagttgcgagattatagtaaacttaaataccatcatgtatacccaacagtttatgctaataaatgcaatatttttcgaaactaaaccttggatcccatataaaatgattggaaataaaaatacctaattataatatttgaatttcagTGGCAGCAGAA**TCC**GGATGCGATTCGGGACATCTTATTCAATCATTCGAAGAAGCTCGCCGTCTCGATAAGATTAATGAAAGAATGCCACCAACAATGGCAACACCACCAGCACAATCACCAAGTCAATCACCAATTCCGTCACGACAAACGACACCTCAACCACCACAAAATGGTCCATCAAATAGCTAAtagtcatagttcatatggcatatcatcttcaaaagtcgttagagtttggaaactaacacgcttgaatacgtaatcttcttcttcttttaattattttttttatctaaaaactttcaaccagctagatgcaaatttccatctccgaaaactatttgcc

mutant sequence (-778bp deletion + 1 synonymous mutation)

TCGACACAAGATTCGAGCAATTGGAAAGATGGCAAGAGCATTCTCAGTTCTTCGgtaagttgattaatttttttcaattttaaaccgaatttgtatgaatttaaatagaaagtttttttctattatagaatgagcattttttttagttaacttttatttatttttgataacatatatcttaatttaatgtttctataaaaaaacaacatacttaaagcatgattttgacaatttttgttaattaatcatttttgattatttttttttcaaagttcataactctttatgtggtttttcatattttcagCGAGGAGAGCGAAAGTGTTCTCGCACTGAAAGGACTTACACCAACCGGTGCACTTCCAATGGGAACACTTCAAGGTGGATCACGTGGTGTCCGAGAAG**-**TGGCAGCAGAA**TCG**GGATGCGATTCGGGACATCTTATTCAATCATTCGAAGAAGCTCGCCGTCTCGATAAGATTAATGAAAGAATGCCACCAACAATGGCAACACCACCAGCACAATCACCAAGTCAATCACCAATTCCGTCACGACAAACGACACCTCAACCACCACAAAATGGTCCATCAAATAGCTAAtagtcatagttcatatggcatatcatcttcaaaagtcgttagagtttggaaactaacacgcttgaatacgtaatcttcttcttcttttaattattttttttatctaaaaactttcaaccagctagatgcaaatttccatctccgaaaactatttgcc

***tax-6(syb1384) (Δex13a)***

wild type sequence

gacccgtttcctctaactcaacctctatcattttcggtgacggttggcacattgcaaaatcccaaatttttaccccctttgccctccccatattcacactatttacaagtagtaatttttcacctattagccaatatatatgtacatggctttcattttatttttgacatttttctaacttcattcggggatttctattgattggtaatgtggttgccagtgaatggtgtcaccccttttgaagttgttgccatgtcatttgtaaatgttgacggttcttttttaaaaattaatttttcagGTGGAGTGGGGTCTGCAAGAAAAGAGGTTATTCGACACAAGATTCGAGCAATTGGAAAGATGGCAAGAGCATTCTCAGTTCTTCGgtaagttgattaatttttttcaattttaaaccgaatttgtatgaatttaaatagaaagtttttttctattatagaatgagcattttttttagttaacttttatttatttttgataacatatatcttaatttaatgtttctataaaaaaacaacatacttaaagcatgattttgacaatttttgttaattaatcatttttgattatttttttttcaaagttcataactctttatgtggtttttcatattttcagCGAGGAGAGCGAAAGTGTTCTCGCACTGAAAGGACTTACACCAACCGGTGCACTTCCAATGGGAACACTTCAAGGTGGATCACGTGGTGTCCGAGAAGgtaatgtttgtcctgatgtctcttcgatcctgatccgtccaaaatcgtttaaagtccgtcaaaaagtgtgtgtacttttctctaatcgaaatgcaactatacttaccgagttaactttcgaatcaaatgatattttctacgaaaaaacaataattcaatagaaaaaaggaaagagaaaaacgtagtaccacgctttttaacagACATGAAAGCGAGTCA**GTG**CTGCAGTTGAAGGGCCTGAATGCAGGTGGCAAGCTGCCACAAGGGGCTCTTTCGGAAGGACGGACAGGATTGAATGCGGCTTATAGAATTGAACAGTCAGgtctgaataataaaaattatctaaattatctatggtctgcaaatgtttaaaacatctgattctacaaaagtatggtttttttaactgaaagaacattgcttttggggcaacaccgacgttttcgttttcgaaaatgtttttaattatttttcttttcaaaattacaactttaaagttgctaaaaaaaaacaaatttgattagacaaccaggaatgaagaaaaatctaagcatctgcattcgattttttttcttttttgtgacgagacccattcaaatagccacgtgcgactttaaagtattaatttcgaaagttgcgagattatagtaaacttaaataccatcatgtatacccaacagtttatgctaataaatgcaatatttttcgaaactaaaccttggatcccatataaaatgattggaaataaaaatacctaattataatatttgaatttcagTGGCAGCAGAATCCGGATGCGATTCGGGACAT

mutant sequence (-554bp deletion & 1 synonymous mutation)

gacccgtttcctctaactcaacctctatcattttcggtgacggttggcacattgcaaaatcccaaatttttaccccctttgccctccccatattcacactatttacaagtagtaatttttcacctattagccaatatatatgtacatggctttcattttatttttgacatttttctaacttcattcggggatttctattgattggtaatgtggttgccagtgaatggtgtcaccccttttgaagttgttgccatgtcatttgtaaatgttgacggttcttttttaaaaattaatttttcagGTGGAGTGGGGTCTGCAAGAAAAGAGGTTATTCGACACAAGATTCGAGCAATTGGAAAGATGGCAAGAGCATTCTCAGTTCTTCG-ACATGAAAGCGAGTCA**GTC**CTGCAGTTGAAGGGCCTGAATGCAGGTGGCAAGCTGCCACAAGGGGCTCTTTCGGAAGGACGGACAGGATTGAATGCGGCTTATAGAATTGAACAGTCAGgtctgaataataaaaattatctaaattatctatggtctgcaaatgtttaaaacatctgattctacaaaagtatggtttttttaactgaaagaacattgcttttggggcaacaccgacgttttcgttttcgaaaatgtttttaattatttttcttttcaaaattacaactttaaagttgctaaaaaaaaacaaatttgattagacaaccaggaatgaagaaaaatctaagcatctgcattcgattttttttcttttttgtgacgagacccattcaaatagccacgtgcgactttaaagtattaatttcgaaagttgcgagattatagtaaacttaaataccatcatgtatacccaacagtttatgctaataaatgcaatatttttcgaaactaaaccttggatcccatataaaatgattggaaataaaaatacctaattataatatttgaatttcagTGGCAGCAGAATCCGGATGCGATTCGGGACAT

***tax-6(syb1396) (tax-6bc S443D)***

wild type sequence

attttcagCGAGGAGAGCGAAAGTGTTCTCGCACTGAAAGGACTTACACCAACCGGTGCACTTCCAATGGGAACACTTCAAGGTGGATCACGTGGTGTCCGAGAAGgtaatgtttgtcctgatgtctcttcgatcctgatccgtccaaaatcgtttaaagtccgtcaaaaagtgtgtgtacttttctctaatcgaaatgcaactatacttaccgagttaactttcgaatcaaatgatattttctacgaaaaaacaataattcaatagaaaaaaggaaagagaaaaacgtagtaccacgctttttaacagACATGAA**AGC**GAGTCA**GTG**CTGCAGTTGAAGGGCCTGAATGCAGGTGGCAAGCTGCCACAAGGGGCTCTTTCGGAAGGACGGACAGGATTGAATGCGGCTTATAGAATTGAACAGTCAGgtctgaataataaaaattatctaaattatctatggtctgcaaatgtttaaaacatctgattctacaaaagtatggtttttttaactgaaagaacattgcttttggggcaacaccgacgttttcgttttcgaaaatgtttttaattatttttcttttcaaaattacaactttaaagttgctaaaaaaaaacaaatttgattagacaaccaggaatgaagaaaaatctaagcatctgcattcgattttttttcttttttgtgacgagacccattcaaatagccacg

mutant sequence (1 missense + 1 synonymous mutation)

attttcagCGAGGAGAGCGAAAGTGTTCTCGCACTGAAAGGACTTACACCAACCGGTGCACTTCCAATGGGAACACTTCAAGGTGGATCACGTGGTGTCCGAGAAGgtaatgtttgtcctgatgtctcttcgatcctgatccgtccaaaatcgtttaaagtccgtcaaaaagtgtgtgtacttttctctaatcgaaatgcaactatacttaccgagttaactttcgaatcaaatgatattttctacgaaaaaacaataattcaatagaaaaaaggaaagagaaaaacgtagtaccacgctttttaacagACATGAA**GAT**GAGTCA**GTA**CTGCAGTTGAAGGGCCTGAATGCAGGTGGCAAGCTGCCACAAGGGGCTCTTTCGGAAGGACGGACAGGATTGAATGCGGCTTATAGAATTGAACAGTCAGgtctgaataataaaaattatctaaattatctatggtctgcaaatgtttaaaacatctgattctacaaaagtatggtttttttaactgaaagaacattgcttttggggcaacaccgacgttttcgttttcgaaaatgtttttaattatttttcttttcaaaattacaactttaaagttgctaaaaaaaaacaaatttgattagacaaccaggaatgaagaaaaatctaagcatctgcattcgattttttttcttttttgtgacgagacccattcaaatagccacg

***tax-6(syb1389) (13a-stop)***

wild type sequence

tttttcagGTGGAGTGGGGTCTGCAAGAAAAGAGGTTATTCGACACAAGATTCGAGCAATTGGAAAGATGGCAAGAGCATTCTCAGTTCTTCGgtaagttgattaatttttttcaattttaaaccgaatttgtatgaatttaaatagaaagtttttttctattatagaatgagcattttttttagttaacttttatttatttttgataacatatatcttaatttaatgtttctataaaaaaacaacatacttaaagcatgattttgacaatttttgttaattaatcatttttgattatttttttttcaaagttcataactctttatgtggtttttcatattttcagCGAGGAG**AGC**GAAAGTGTTCTCGCACTGAAAGGACTTACACCAACCGGT**GCA**CTTCCAATGGGAACACTTCAAGGTGGATCACGTGGTGTCCGAGAAGgtaatgtttgtcctgatgtctcttcgatcctgatccgtccaaaatcgtttaaagtccgtcaaaaagtgtgtgtacttttctctaatcgaaatgcaactatacttaccgagttaactttcgaatcaaatgatattttctacgaaaaaacaataattcaatagaaaaaaggaaagagaaaaacgtagtaccacgctttttaacagacatgaaagcgagtcagtgctgcagttgaagggcctgaatgcaggtggcaagctgccacaaggggctctttcggaaggacggacaggattgaa

mutant sequence (1 nonsense + 1 synonymous mutation)

tttttcagGTGGAGTGGGGTCTGCAAGAAAAGAGGTTATTCGACACAAGATTCGAGCAATTGGAAAGATGGCAAGAGCATTCTCAGTTCTTCGgtaagttgattaatttttttcaattttaaaccgaatttgtatgaatttaaatagaaagtttttttctattatagaatgagcattttttttagttaacttttatttatttttgataacatatatcttaatttaatgtttctataaaaaaacaacatacttaaagcatgattttgacaatttttgttaattaatcatttttgattatttttttttcaaagttcataactctttatgtggtttttcatattttcagCGAGGAG**TAG**GAAAGTGTTCTCGCACTGAAAGGACTTACACCAACCGGT**GCG**CTTCCAATGGGAACACTTCAAGGTGGATCACGTGGTGTCCGAGAAGgtaatgtttgtcctgatgtctcttcgatcctgatccgtccaaaatcgtttaaagtccgtcaaaaagtgtgtgtacttttctctaatcgaaatgcaactatacttaccgagttaactttcgaatcaaatgatattttctacgaaaaaacaataattcaatagaaaaaaggaaagagaaaaacgtagtaccacgctttttaacagacatgaaagcgagtcagtgctgcagttgaagggcctgaatgcaggtggcaagctgccacaaggggctctttcggaaggacggacaggattgaa

***tax-6(syb1402) (tax-6bc S443D + Δex13a)***

wild type sequence

ttaaaaattaatttttcagGTGGAGTGGGGTCTGCAAGA**AAA**GAGGTTATTCGACACAAGATTCGAGCAATTGGAAAGATGGCAAGAGCATTCTCAGTTCTTCGgtaagttgattaatttttttcaattttaaaccgaatttgtatgaatttaaatagaaagtttttttctattatagaatgagcattttttttagttaacttttatttatttttgataacatatatcttaatttaatgtttctataaaaaaacaacatacttaaagcatgattttgacaatttttgttaattaatcatttttgattatttttttttcaaagttcataactctttatgtggtttttcatattttcagCGAGGAGAGCGAAAGTGTTCTCGCACTGAAAGGACTTACACCAACCGGTGCACTTCCAATGGGAACACTTCAAGGTGGATCACGTGGTGTCCGAGAAGgtaatgtttgtcctgatgtctcttcgatcctgatccgtccaaaatcgtttaaagtccgtcaaaaagtgtgtgtacttttctctaatcgaaatgcaactatacttaccgagttaactttcgaatcaaatgatattttctacgaaaaaacaataattcaatagaaaaaaggaaagagaaaaacgtagtaccacgctttttaacagACATGAA**AGC**GAGTCAGTGCTGCAGTTGAAG**GGC**CTGAATGCAGGTGGCAAGCTGCCACAAGGGGCTCTTTCGGAAGGACGGACAGGATTGAATGCGGCTTATAGAATTGAACAGTCAGgtctgaataataaaaattatctaaattatctatggtctgcaaatgtttaaaacatctgattctacaaaagtatggtttttttaactgaaagaacattgcttttggggc

mutant sequence (-554bp deletion, 1 missense mutation & 2 synonymous mutations)

ttaaaaattaatttttcagGTGGAGTGGGGTCTGCAAGA**AAG**GAGGTTATTCGACACAAGATTCGAGCAATTGGAAAGATGGCAAGAGCATTCTCAGTTCTTCG-ACATGAA**GAC**GAGTCAGTGCTGCAGTTGAAG**GGT**CTGAATGCAGGTGGCAAGCTGCCACAAGGGGCTCTTTCGGAAGGACGGACAGGATTGAATGCGGCTTATAGAATTGAACAGTCAGgtctgaataataaaaattatctaaattatctatggtctgcaaatgtttaaaacatctgattctacaaaagtatggtttttttaactgaaagaacattgcttttggggc

***tax-6(syb1391) (tax-6bc S443A + Δex13a)***

wild type sequence

ctcacttggttttgattccccaccctaaaaattagttactaattggaagaaatgggtgtctttattgggatttttaggggtacggtataagaaatgagaaagtggaaatttccccaaacctgtgagaaaagaccttgaagtgtatattttttctatacataatattttctcaaaaaggttactttatattttattagtatattacggtatatttgaaaaattattatgaaattgtaaaagtagaattgatgtttactcgacaagaagattttttaaactatgagttttttggctattcttacggaacataatcttggaacgaaataagaaaatgttaacaatcgaacaagtttagttttttgtataaaaaattgtagttaaattaactagacaaataaattttcttttccaagagcataaaacttctttaaaactatttacaacaaaaactaccgtaatcccttcaaatcccaatgggacccgtttcctctaactcaacctctatcattttcggtgacggttggcacattgcaaaatcccaaatttttaccccctttgccctccccatattcacactatttacaagtagtaatttttcacctattagccaatatatatgtacatggctttcattttatttttgacatttttctaacttcattcggggatttctattgattggtaatgtggttgccagtgaatggtgtcaccccttttgaagttgttgccatgtcatttgtaaatgttgacggttcttttttaaaaattaatttttcagGTGGAGTGGGGTCTGCAAGA**AAA**GAGGTTATTCGACACAAGATTCGAGCAATTGGAAAGATGGCAAGAGCATTCTCAGTTCTTCGgtaagttgattaatttttttcaattttaaaccgaatttgtatgaatttaaatagaaagtttttttctattatagaatgagcattttttttagttaacttttatttatttttgataacatatatcttaatttaatgtttctataaaaaaacaacatacttaaagcatgattttgacaatttttgttaattaatcatttttgattatttttttttcaaagttcataactctttatgtggtttttcatattttcagCGAGGAGAGCGAAAGTGTTCTCGCACTGAAAGGACTTACACCAACCGGTGCACTTCCAATGGGAACACTTCAAGGTGGATCACGTGGTGTCCGAGAAGgtaatgtttgtcctgatgtctcttcgatcctgatccgtccaaaatcgtttaaagtccgtcaaaaagtgtgtgtacttttctctaatcgaaatgcaactatacttaccgagttaactttcgaatcaaatgatattttctacgaaaaaacaataattcaatagaaaaaaggaaagagaaaaacgtagtaccacgctttttaacagACATGAA**AGC**GAGTCAGTGCTGCAGTTGAAG**GGC**CTGAATGCAGGTGGCAAGCTGCCACAAGGGGCTCTTTCGGAAGGACGGACAGGATTGAATGCGGCTTATAGAATTGAACAGTCAGgtctgaataataaaaattatctaaattatctatggtctgcaaatgtttaaaacatctgattctacaaaagtatggtttttttaactgaaagaacattgcttttggggcaacaccgacgttttcgttttcgaaaatgtttttaattatttttcttttcaaaattacaactttaaagttgctaaaaaaaaacaaatttgattagacaaccaggaatgaaga

mutant sequence (-554bp deletion, 1 missense mutation & 2 synonymous mutations)

ctcacttggttttgattccccaccctaaaaattagttactaattggaagaaatgggtgtctttattgggatttttaggggtacggtataagaaatgagaaagtggaaatttccccaaacctgtgagaaaagaccttgaagtgtatattttttctatacataatattttctcaaaaaggttactttatattttattagtatattacggtatatttgaaaaattattatgaaattgtaaaagtagaattgatgtttactcgacaagaagattttttaaactatgagttttttggctattcttacggaacataatcttggaacgaaataagaaaatgttaacaatcgaacaagtttagttttttgtataaaaaattgtagttaaattaactagacaaataaattttcttttccaagagcataaaacttctttaaaactatttacaacaaaaactaccgtaatcccttcaaatcccaatgggacccgtttcctctaactcaacctctatcattttcggtgacggttggcacattgcaaaatcccaaatttttaccccctttgccctccccatattcacactatttacaagtagtaatttttcacctattagccaatatatatgtacatggctttcattttatttttgacatttttctaacttcattcggggatttctattgattggtaatgtggttgccagtgaatggtgtcaccccttttgaagttgttgccatgtcatttgtaaatgttgacggttcttttttaaaaattaatttttcagGTGGAGTGGGGTCTGCAAGA**AAG**GAGGTTATTCGACACAAGATTCGAGCAATTGGAAAGATGGCAAGAGCATTCTCAGTTCTTCG-ACATGAA**GCC**GAGTCAGTGCTGCAGTTGAAG**GGT**CTGAATGCAGGTGGCAAGCTGCCACAAGGGGCTCTTTCGGAAGGACGGACAGGATTGAATGCGGCTTATAGAATTGAACAGTCAGgtctgaataataaaaattatctaaattatctatggtctgcaaatgtttaaaacatctgattctacaaaagtatggtttttttaactgaaagaacattgcttttggggcaacaccgacgttttcgttttcgaaaatgtttttaattatttttcttttcaaaattacaactttaaagttgctaaaaaaaaacaaatttgattagacaaccaggaatgaaga

***tax-6(syb2325) (tax-6a S443A)***

wild type sequence

tatgtggtttttcatattttcagCGAGGAG**AGC**GAAAGTGTTCTCGCACTGAAAGGACTTACACCAACCGGT**GCA**

mutant sequence (1 missense mutation & 1 synonymous mutation)

tatgtggtttttcatattttcagCGAGGAG**GCC**GAAAGTGTTCTCGCACTGAAAGGACTTACACCAACCGGT**GCG**

***tax-6(syb2280) (tax-6a S443D)***

wild type sequence

tatgtggtttttcatattttcagCGAGGAG**AGC**GAAAGTGTTCTCGCACTGAAAGGACTTACACCAACCGGT**GCA**

mutant sequence (1 missense mutation & 1 synonymous mutation)

tatgtggtttttcatattttcagCGAGGAG**GAT**GAAAGTGTTCTCGCACTGAAAGGACTTACACCAACCGGT**GCG**

***tax-6(syb2324) (tax-6a S443A+ Δex13bc)***

wild type sequence

GAGCAATTGGAAAGATGGCAAGAGCATTCTCAGTTCTTCGgtaagttgattaatttttttcaattttaaaccgaatttgtatgaatttaaatagaaagtttttttctattatagaatgagcattttttttagttaacttttatttatttttgataacatatatcttaatttaatgtttctataaaaaaacaacatacttaaagcatgattttgacaatttttgttaattaatcatttttgattatttttttttcaaagttcataactctttatgtggtttttcatattttcagCGAGGAG**AGC**GAAAGTGTTCTCGCACTGAAAGGACTTACACCAACCGGT**GCA**CTTCCAATGGGAACACTTCAAGGTGGATCACGTGGTGTCCGAGAAGgtaatgtttgtcctgatgtctcttcgatcctgatccgtccaaaatcgtttaaagtccgtcaaaaagtgtgtgtacttttctctaatcgaaatgcaactatacttaccgagttaactttcgaatcaaatgatattttctacgaaaaaacaataattcaatagaaaaaaggaaagagaaaaacgtagtaccacgctttttaacagACATGAAAGCGAGTCAGTGCTGCAGTTGAAGGGCCTGAATGCAGGTGGCAAGCTGCCACAAGGGGCTCTTTCGGAAGGACGGACAGGATTGAATGCGGCTTATAGAATTGAACAGTCAGgtctgaataataaaaattatctaaattatctatggtctgcaaatgtttaaaacatctgattctacaaaagtatggtttttttaactgaaagaacattgcttttggggcaacaccgacgttttcgttttcgaaaatgtttttaattatttttcttttcaaaattacaactttaaagttgctaaaaaaaaacaaatttgattagacaaccaggaatgaagaaaaatctaagcatctgcattcgattttttttcttttttgtgacgagacccattcaaatagccacgtgcgactttaaagtattaatttcgaaagttgcgagattatagtaaacttaaataccatcatgtatacccaacagtttatgctaataaatgcaatatttttcgaaactaaaccttggatcccatataaaatgattggaaataaaaatacctaattataatatttgaatttcagTGGCAGCAGAA**TCC**GGATGCGATTCGGGACATCTTATTCAATCATTCGAAGAAGCTCGCCGTCTCGATAAGATTAATGAAAGAATGCCACCAACAATGGCAACACCACCAGCACAATCACCAAGTCAATCACCAATTCCGTCACGACAAACGACACCTCAACCACCACAAAATGGTCCATCAAATAGCTAAtagtcatagttcatatggcat

mutant sequence (-778 bp deletion, 1 missense mutation & 2 synonymous mutations)

GAGCAATTGGAAAGATGGCAAGAGCATTCTCAGTTCTTCGgtaagttgattaatttttttcaattttaaaccgaatttgtatgaatttaaatagaaagtttttttctattatagaatgagcattttttttagttaacttttatttatttttgataacatatatcttaatttaatgtttctataaaaaaacaacatacttaaagcatgattttgacaatttttgttaattaatcatttttgattatttttttttcaaagttcataactctttatgtggtttttcatattttcagCGAGGAG**GCC**GAAAGTGTTCTCGCACTGAAAGGACTTACACCAACCGGT**GCG**CTTCCAATGGGAACACTTCAAGGTGGATCACGTGGTGTCCGAGAAG-TGGCAGCAGAA**TCG**GGATGCGATTCGGGACATCTTATTCAATCATTCGAAGAAGCTCGCCGTCTCGATAAGATTAATGAAAGAATGCCACCAACAATGGCAACACCACCAGCACAATCACCAAGTCAATCACCAATTCCGTCACGACAAACGACACCTCAACCACCACAAAATGGTCCATCAAATAGCTAAtagtcatagttcatatggcat

***tax-6(syb2371) (tax-6a S443D+ Δex13bc)***

wild type sequence

GAGCAATTGGAAAGATGGCAAGAGCATTCTCAGTTCTTCGgtaagttgattaatttttttcaattttaaaccgaatttgtatgaatttaaatagaaagtttttttctattatagaatgagcattttttttagttaacttttatttatttttgataacatatatcttaatttaatgtttctataaaaaaacaacatacttaaagcatgattttgacaatttttgttaattaatcatttttgattatttttttttcaaagttcataactctttatgtggtttttcatattttcagCGAGGAG**AGC**GAAAGTGTTCTCGCACTGAAAGGACTTACACCAACCGGT**GCA**CTTCCAATGGGAACACTTCAAGGTGGATCACGTGGTGTCCGAGAAGgtaatgtttgtcctgatgtctcttcgatcctgatccgtccaaaatcgtttaaagtccgtcaaaaagtgtgtgtacttttctctaatcgaaatgcaactatacttaccgagttaactttcgaatcaaatgatattttctacgaaaaaacaataattcaatagaaaaaaggaaagagaaaaacgtagtaccacgctttttaacagACATGAAAGCGAGTCAGTGCTGCAGTTGAAGGGCCTGAATGCAGGTGGCAAGCTGCCACAAGGGGCTCTTTCGGAAGGACGGACAGGATTGAATGCGGCTTATAGAATTGAACAGTCAGgtctgaataataaaaattatctaaattatctatggtctgcaaatgtttaaaacatctgattctacaaaagtatggtttttttaactgaaagaacattgcttttggggcaacaccgacgttttcgttttcgaaaatgtttttaattatttttcttttcaaaattacaactttaaagttgctaaaaaaaaacaaatttgattagacaaccaggaatgaagaaaaatctaagcatctgcattcgattttttttcttttttgtgacgagacccattcaaatagccacgtgcgactttaaagtattaatttcgaaagttgcgagattatagtaaacttaaataccatcatgtatacccaacagtttatgctaataaatgcaatatttttcgaaactaaaccttggatcccatataaaatgattggaaataaaaatacctaattataatatttgaatttcagTGGCAGCAGAA**TCC**GGATGCGATTCGGGACATCTTATTCAATCATTCGAAGAAGCTCGCCGTCTCGATAAGATTAATGAAAGAATGCCACCAACAATGGCAACACCACCAGCACAATCACCAAGTCAATCACCAATTCCGTCACGACAAACGACACCTCAACCACCACAAAATGGTCCATCAAATAGCTAAtagtcatagttcatatggcat

mutant sequence (-778 bp deletion, 1 missense mutation & 2 synonymous mutations)

GAGCAATTGGAAAGATGGCAAGAGCATTCTCAGTTCTTCGgtaagttgattaatttttttcaattttaaaccgaatttgtatgaatttaaatagaaagtttttttctattatagaatgagcattttttttagttaacttttatttatttttgataacatatatcttaatttaatgtttctataaaaaaacaacatacttaaagcatgattttgacaatttttgttaattaatcatttttgattatttttttttcaaagttcataactctttatgtggtttttcatattttcagCGAGGAG**GAT**GAAAGTGTTCTCGCACTGAAAGGACTTACACCAACCGGT**GCG**CTTCCAATGGGAACACTTCAAGGTGGATCACGTGGTGTCCGAGAAG-TGGCAGCAGAA**TCG**GGATGCGATTCGGGACATCTTATTCAATCATTCGAAGAAGCTCGCCGTCTCGATAAGATTAATGAAAGAATGCCACCAACAATGGCAACACCACCAGCACAATCACCAAGTCAATCACCAATTCCGTCACGACAAACGACACCTCAACCACCACAAAATGGTCCATCAAATAGCTAAtagtcatagttcatatggcat

***tax-6(syb2282) (13a stop + Δex13bc)***

wild type sequence

GAGCAATTGGAAAGATGGCAAGAGCATTCTCAGTTCTTCGgtaagttgattaatttttttcaattttaaaccgaatttgtatgaatttaaatagaaagtttttttctattatagaatgagcattttttttagttaacttttatttatttttgataacatatatcttaatttaatgtttctataaaaaaacaacatacttaaagcatgattttgacaatttttgttaattaatcatttttgattatttttttttcaaagttcataactctttatgtggtttttcatattttcagCGAGGAG**AGC**GAAAGTGTTCTCGCACTGAAAGGACTTACACCAACCGGT**GCA**CTTCCAATGGGAACACTTCAAGGTGGATCACGTGGTGTCCGAGAAGgtaatgtttgtcctgatgtctcttcgatcctgatccgtccaaaatcgtttaaagtccgtcaaaaagtgtgtgtacttttctctaatcgaaatgcaactatacttaccgagttaactttcgaatcaaatgatattttctacgaaaaaacaataattcaatagaaaaaaggaaagagaaaaacgtagtaccacgctttttaacagACATGAAAGCGAGTCAGTGCTGCAGTTGAAGGGCCTGAATGCAGGTGGCAAGCTGCCACAAGGGGCTCTTTCGGAAGGACGGACAGGATTGAATGCGGCTTATAGAATTGAACAGTCAGgtctgaataataaaaattatctaaattatctatggtctgcaaatgtttaaaacatctgattctacaaaagtatggtttttttaactgaaagaacattgcttttggggcaacaccgacgttttcgttttcgaaaatgtttttaattatttttcttttcaaaattacaactttaaagttgctaaaaaaaaacaaatttgattagacaaccaggaatgaagaaaaatctaagcatctgcattcgattttttttcttttttgtgacgagacccattcaaatagccacgtgcgactttaaagtattaatttcgaaagttgcgagattatagtaaacttaaataccatcatgtatacccaacagtttatgctaataaatgcaatatttttcgaaactaaaccttggatcccatataaaatgattggaaataaaaatacctaattataatatttgaatttcagTGGCAGCAGAA**TCC**GGATGCGATTCGGGACATCTTATTCAATCATTCGAAGAAGCTCGCCGTCTCGATAAGATTAATGAAAGAATGCCACCAACAATGGCAACACCACCAGCACAATCACCAAGTCAATCACCAATTCCGTCACGACAAACGACACCTCAACCACCACAAAATGGTCCATCAAATAGCTAAtagtcatagttcatatggcat

mutant sequence (-778 bp deletion, 1 nonsense mutation & 2 synonymous mutations)

GAGCAATTGGAAAGATGGCAAGAGCATTCTCAGTTCTTCGgtaagttgattaatttttttcaattttaaaccgaatttgtatgaatttaaatagaaagtttttttctattatagaatgagcattttttttagttaacttttatttatttttgataacatatatcttaatttaatgtttctataaaaaaacaacatacttaaagcatgattttgacaatttttgttaattaatcatttttgattatttttttttcaaagttcataactctttatgtggtttttcatattttcagCGAGGAG**TAG**GAAAGTGTTCTCGCACTGAAAGGACTTACACCAACCGGT**GCG**CTTCCAATGGGAACACTTCAAGGTGGATCACGTGGTGTCCGAGAAG-TGGCAGCAGAA**TCG**GGATGCGATTCGGGACATCTTATTCAATCATTCGAAGAAGCTCGCCGTCTCGATAAGATTAATGAAAGAATGCCACCAACAATGGCAACACCACCAGCACAATCACCAAGTCAATCACCAATTCCGTCACGACAAACGACACCTCAACCACCACAAAATGGTCCATCAAATAGCTAAtagtcatagttcatatggcat

***tax-6(syb2317) (tax-6a S443A + tax-6bc S443A)***

wild type sequence

tatgtggtttttcatattttcagCGAGGAG**AGC**GAAAGTGTTCTCGCACTGAAAGGACTTACACCAACCGGT**GCA**CTTCCAATGGGAACACTTCAAGGTGGATCACGTGGTGTCCGAGAAGgtaatgtttgtcctgatgtctcttcgatcctgatccgtccaaaatcgtttaaagtccgtcaaaaagtgtgtgtacttttctctaatcgaaatgcaactatacttaccgagttaactttcgaatcaaatgatattttctacgaaaaaacaataattcaatagaaaaaaggaaagagaaaaacgtagtaccacgctttttaacagACATGAA**AGC**GAGTCA**GTG**CTGCAGTTGAAGGGCCTGAATGCAGGTGGCAAGCTGCCACAAGGGGCTCTTTCGGAAGGACGGACAGGATTGAATGCGGCTTATAGAATTGAACAGTCAGgtctgaataataaaaattatctaaattatctatggtctgcaaatgtttaaaacatctgattctacaaaagtatggtttttttaactgaaagaacattgcttttggggcaacaccgacgttttcgttttcgaaaatgtttttaattatttttcttttcaaaattacaactttaaagttgctaaaaaaaaacaaatttgattagacaaccaggaatgaagaaaaatctaagcatctgcattcgattttttttcttttttgtgacgagacccattcaaatagccacgtgcgactttaaagtattaatttcgaaagttgcgagattatagtaaacttaaataccatcatgtatacccaacagtttatgctaataaatgcaatatttttcgaaactaaaccttggatcccatataaaatgattggaaataaaaatacctaattataatatttgaatttcagTGGCAGCAGAATCCGGATGCGATTCGGGACATCTTATTCAATCATTCGAAGAAGCTCGCCGTCTCGATAAGATTAATGAAAGAATGCCACCAACAATGGCAACACCACCAGCACAATCACCAAGTCAATCACCAATTCCGTCACGACAAACGACACCTCAACCACCACAAAATGGTCCATCAAATAGCTAAtagtcatagttcatatggcat

mutant sequence (2 missense mutations & 2 synonymous mutations)

tatgtggtttttcatattttcagCGAGGAG**GCC**GAAAGTGTTCTCGCACTGAAAGGACTTACACCAACCGGT**GCG**CTTCCAATGGGAACACTTCAAGGTGGATCACGTGGTGTCCGAGAAGgtaatgtttgtcctgatgtctcttcgatcctgatccgtccaaaatcgtttaaagtccgtcaaaaagtgtgtgtacttttctctaatcgaaatgcaactatacttaccgagttaactttcgaatcaaatgatattttctacgaaaaaacaataattcaatagaaaaaaggaaagagaaaaacgtagtaccacgctttttaacagACATGAA**GCA**GAGTCA**GTA**CTGCAGTTGAAGGGCCTGAATGCAGGTGGCAAGCTGCCACAAGGGGCTCTTTCGGAAGGACGGACAGGATTGAATGCGGCTTATAGAATTGAACAGTCAGgtctgaataataaaaattatctaaattatctatggtctgcaaatgtttaaaacatctgattctacaaaagtatggtttttttaactgaaagaacattgcttttggggcaacaccgacgttttcgttttcgaaaatgtttttaattatttttcttttcaaaattacaactttaaagttgctaaaaaaaaacaaatttgattagacaaccaggaatgaagaaaaatctaagcatctgcattcgattttttttcttttttgtgacgagacccattcaaatagccacgtgcgactttaaagtattaatttcgaaagttgcgagattatagtaaacttaaataccatcatgtatacccaacagtttatgctaataaatgcaatatttttcgaaactaaaccttggatcccatataaaatgattggaaataaaaatacctaattataatatttgaatttcagTGGCAGCAGAATCCGGATGCGATTCGGGACATCTTATTCAATCATTCGAAGAAGCTCGCCGTCTCGATAAGATTAATGAAAGAATGCCACCAACAATGGCAACACCACCAGCACAATCACCAAGTCAATCACCAATTCCGTCACGACAAACGACACCTCAACCACCACAAAATGGTCCATCAAATAGCTAAtagtcatagttcatatggcat

***tax-6(syb2281) (tax-6a S443D + tax-6bc S443D)***

wild type sequence

tatgtggtttttcatattttcagCGAGGAG**AGC**GAAAGTGTTCTCGCACTGAAAGGACTTACACCAACCGGT**GCA**CTTCCAATGGGAACACTTCAAGGTGGATCACGTGGTGTCCGAGAAGgtaatgtttgtcctgatgtctcttcgatcctgatccgtccaaaatcgtttaaagtccgtcaaaaagtgtgtgtacttttctctaatcgaaatgcaactatacttaccgagttaactttcgaatcaaatgatattttctacgaaaaaacaataattcaatagaaaaaaggaaagagaaaaacgtagtaccacgctttttaacagACATGAA**AGC**GAGTCA**GTG**CTGCAGTTGAAGGGCCTGAATGCAGGTGGCAAGCTGCCACAAGGGGCTCTTTCGGAAGGACGGACAGGATTGAATGCGGCTTATAGAATTGAACAGTCAGgtctgaataataaaaattatctaaattatctatggtctgcaaatgtttaaaacatctgattctacaaaagtatggtttttttaactgaaagaacattgcttttggggcaacaccgacgttttcgttttcgaaaatgtttttaattatttttcttttcaaaattacaactttaaagttgctaaaaaaaaacaaatttgattagacaaccaggaatgaagaaaaatctaagcatctgcattcgattttttttcttttttgtgacgagacccattcaaatagccacgtgcgactttaaagtattaatttcgaaagttgcgagattatagtaaacttaaataccatcatgtatacccaacagtttatgctaataaatgcaatatttttcgaaactaaaccttggatcccatataaaatgattggaaataaaaatacctaattataatatttgaatttcagTGGCAGCAGAATCCGGATGCGATTCGGGACATCTTATTCAATCATTCGAAGAAGCTCGCCGTCTCGATAAGATTAATGAAAGAATGCCACCAACAATGGCAACACCACCAGCACAATCACCAAGTCAATCACCAATTCCGTCACGACAAACGACACCTCAACCACCACAAAATGGTCCATCAAATAGCTAAtagtcatagttcatatggcat

mutant sequence (2 missense mutations & 2 synonymous mutations)

tatgtggtttttcatattttcagCGAGGAG**GAT**GAAAGTGTTCTCGCACTGAAAGGACTTACACCAACCGGT**GCG**CTTCCAATGGGAACACTTCAAGGTGGATCACGTGGTGTCCGAGAAGgtaatgtttgtcctgatgtctcttcgatcctgatccgtccaaaatcgtttaaagtccgtcaaaaagtgtgtgtacttttctctaatcgaaatgcaactatacttaccgagttaactttcgaatcaaatgatattttctacgaaaaaacaataattcaatagaaaaaaggaaagagaaaaacgtagtaccacgctttttaacagACATGAA**GAT**GAGTCA**GTA**CTGCAGTTGAAGGGCCTGAATGCAGGTGGCAAGCTGCCACAAGGGGCTCTTTCGGAAGGACGGACAGGATTGAATGCGGCTTATAGAATTGAACAGTCAGgtctgaataataaaaattatctaaattatctatggtctgcaaatgtttaaaacatctgattctacaaaagtatggtttttttaactgaaagaacattgcttttggggcaacaccgacgttttcgttttcgaaaatgtttttaattatttttcttttcaaaattacaactttaaagttgctaaaaaaaaacaaatttgattagacaaccaggaatgaagaaaaatctaagcatctgcattcgattttttttcttttttgtgacgagacccattcaaatagccacgtgcgactttaaagtattaatttcgaaagttgcgagattatagtaaacttaaataccatcatgtatacccaacagtttatgctaataaatgcaatatttttcgaaactaaaccttggatcccatataaaatgattggaaataaaaatacctaattataatatttgaatttcagTGGCAGCAGAATCCGGATGCGATTCGGGACATCTTATTCAATCATTCGAAGAAGCTCGCCGTCTCGATAAGATTAATGAAAGAATGCCACCAACAATGGCAACACCACCAGCACAATCACCAAGTCAATCACCAATTCCGTCACGACAAACGACACCTCAACCACCACAAAATGGTCCATCAAATAGCTAAtagtcatagttcatatggcat

***tax-6(syb2354) (13a-stop + 13bc-stop)***

wild type sequence

tatgtggtttttcatattttcagCGAGGAG**AGC**GAAAGTGTTCTCGCACTGAAAGGACTTACACCAACCGGT**GCA**CTTCCAATGGGAACACTTCAAGGTGGATCACGTGGTGTCCGAGAAGgtaatgtttgtcctgatgtctcttcgatcctgatccgtccaaaatcgtttaaagtccgtcaaaaagtgtgtgtacttttctctaatcgaaatgcaactatacttaccgagttaactttcgaatcaaatgatattttctacgaaaaaacaataattcaatagaaaaaaggaaagagaaaaacgtagtaccacgctttttaacagACATGAA**AGC**GAGTCA**GTG**CTGCAGTTGAAGGGCCTGAATGCAGGTGGCAAGCTGCCACAAGGGGCTCTTTCGGAAGGACGGACAGGATTGAATGCGGCTTATAGAATTGAACAGTCAGgtctgaataataaaaattatctaaattatctatggtctgcaaatgtttaaaacatctgattctacaaaagtatggtttttttaactgaaagaacattgcttttggggcaacaccgacgttttcgttttcgaaaatgtttttaattatttttcttttcaaaattacaactttaaagttgctaaaaaaaaacaaatttgattagacaaccaggaatgaagaaaaatctaagcatctgcattcgattttttttcttttttgtgacgagacccattcaaatagccacgtgcgactttaaagtattaatttcgaaagttgcgagattatagtaaacttaaataccatcatgtatacccaacagtttatgctaataaatgcaatatttttcgaaactaaaccttggatcccatataaaatgattggaaataaaaatacctaattataatatttgaatttcagTGGCAGCAGAATCCGGATGCGATTCGGGACATCTTATTCAATCATTCGAAGAAGCTCGCCGTCTCGATAAGATTAATGAAAGAATGCCACCAACAATGGCAACACCACCAGCACAATCACCAAGTCAATCACCAATTCCGTCACGACAAACGACACCTCAACCACCACAAAATGGTCCATCAAATAGCTAAtagtcatagttcatatggcat

mutant sequence (2 missense mutations & 2 synonymous mutations)

tatgtggtttttcatattttcagCGAGGAG**TAG**GAAAGTGTTCTCGCACTGAAAGGACTTACACCAACCGGT**GCG**CTTCCAATGGGAACACTTCAAGGTGGATCACGTGGTGTCCGAGAAGgtaatgtttgtcctgatgtctcttcgatcctgatccgtccaaaatcgtttaaagtccgtcaaaaagtgtgtgtacttttctctaatcgaaatgcaactatacttaccgagttaactttcgaatcaaatgatattttctacgaaaaaacaataattcaatagaaaaaaggaaagagaaaaacgtagtaccacgctttttaacagACATGAA**TAG**GAGTCA**GTA**CTGCAGTTGAAGGGCCTGAATGCAGGTGGCAAGCTGCCACAAGGGGCTCTTTCGGAAGGACGGACAGGATTGAATGCGGCTTATAGAATTGAACAGTCAGgtctgaataataaaaattatctaaattatctatggtctgcaaatgtttaaaacatctgattctacaaaagtatggtttttttaactgaaagaacattgcttttggggcaacaccgacgttttcgttttcgaaaatgtttttaattatttttcttttcaaaattacaactttaaagttgctaaaaaaaaacaaatttgattagacaaccaggaatgaagaaaaatctaagcatctgcattcgattttttttcttttttgtgacgagacccattcaaatagccacgtgcgactttaaagtattaatttcgaaagttgcgagattatagtaaacttaaataccatcatgtatacccaacagtttatgctaataaatgcaatatttttcgaaactaaaccttggatcccatataaaatgattggaaataaaaatacctaattataatatttgaatttcagTGGCAGCAGAATCCGGATGCGATTCGGGACATCTTATTCAATCATTCGAAGAAGCTCGCCGTCTCGATAAGATTAATGAAAGAATGCCACCAACAATGGCAACACCACCAGCACAATCACCAAGTCAATCACCAATTCCGTCACGACAAACGACACCTCAACCACCACAAAATGGTCCATCAAATAGCTAAtagtcatagttcatatggcat
