# Supplementary material for: Conserved Calcineurin A splice variants regulate both constitutive and experience-dependent behaviors through tissue-specific signaling
Source: PLoS Genet. 2025 Sep 26;21(9):e1011884. doi: 10.1371/journal.pgen.1011884 (PMC12503234; doi:10.1371/journal.pgen.1011884)
Supplement: S3 Fig — (A) tax-6 gene description with isoform definition as in Fig 1A (replicated panel). (B) Number of transcripts per million (TPM) measured for individual neuron types in the CeNGEN analysis. Generated on October 10, 2024 https://cengen.shinyapps.io/isoform_compare/ (PDF) [file pgen.1011884.s003.pdf]

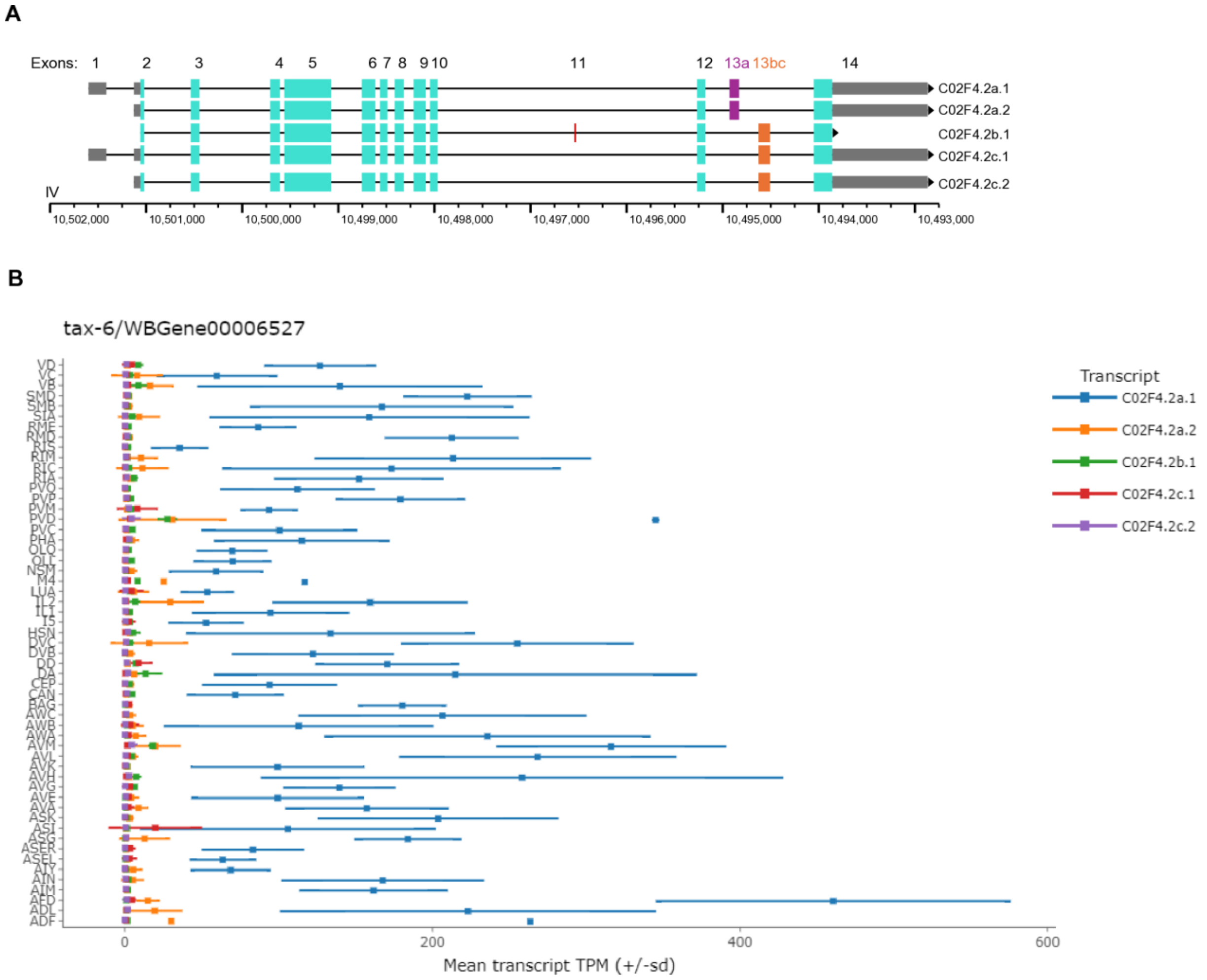

S3 Fig. *tax-6* isoform expression in individual neuron types.

(A) *tax-6* gene description with isoform definition as in Fig 1A (replicated panel).

(B) Number of transcripts per million (TPM) measured for individual neuron types in the CeNGEN analysis. Generated on October 10, 2024 https://cengen.shinyapps.io/isoform_compare/
